# Mindfulness-Based Stress Reduction Alleviates Depression, Anxiety, and Internalized Stigma Compared With Treatment-as-Usual Among Head and Neck Cancer Patients: Findings From a Randomized Controlled Trial

**DOI:** 10.1155/da/7499120

**Published:** 2025-09-11

**Authors:** Zheng Zhang, Qingqin Zhang, Ping Lu, Nurul Izzah Shari, Nik Ruzyanei Nik Jaafar, Mohd Razif Mohamad Yunus, Qiyue Qiu, Fuad Ismail, Nor Faizah Ab Muin, Mohammad Farris Iman Leong Bin Abdullah

**Affiliations:** ^1^Department of Oncology, First Affiliated Hospital, Xinxiang Medical University, Xinxiang, Henan, China; ^2^Department of Community Health, Advanced Medical and Dental Institute, Universiti Sains Malaysia, Kepala Batas, Pulau Pinang, Malaysia; ^3^School of Human Resource Development and Psychology, Faculty of Social Sciences and Humanities, Universiti Teknologi Malaysia, Skudai, Johor Bahru, Johor, Malaysia; ^4^Department of Psychiatry, Universiti Kebangsaan Malaysia Medical Center, Universiti Kebangsaan Malaysia, Cheras, Kuala Lumpur, Malaysia; ^5^Department of Otorhinolaryngology, Universiti Kebangsaan Malaysia Medical Center, Universiti Kebangsaan Malaysia, Cheras, Kuala Lumpur, Malaysia; ^6^School of Pharmaceutical Sciences, Universiti Sains Malaysia, Minden, Pulau Pinang, Malaysia; ^7^Department of Oncology, Universiti Kebangsaan Malaysia Medical Center, Universiti Kebangsaan Malaysia, Cheras, Kuala Lumpur, Malaysia; ^8^Department of Psychiatry and Mental Health, Faculty of Medicine, Universiti Sultan Zainal Abidin, Kuala Terengganu, Terengganu, Malaysia

**Keywords:** anxiety, depression, head and neck cancer, internalized stigma, MBSR, RCT

## Abstract

**Background:** This study aimed to: (1) compare the rates of change in the severity of depression and anxiety symptoms (primary outcomes) as well as internalized stigma and its components (shame with appearance [SWA], speech and social concerns [SSCs], sense of stigma [SS], and regret [R]; secondary outcomes) between the mindfulness-based stress reduction (MBSR) group and the treatment-as-usual (TAU) control group across three timepoints (*T*_0_ = baseline assessment, prior to intervention; *T*_1_ = postintervention, immediately after completion of intervention or at 8 weeks after commence of intervention; *T*_2_ = follow-up assessment, 12 weeks after completion of intervention), and (2) evaluate the mediating effects of reductions in internalized stigma and its components on the relationship between MBSR and the severity of depression and anxiety symptoms among head and neck cancer (HNC) patients.

**Methods:** This multicenter, two-armed, parallel, and double-blind randomized controlled trial (RCT) recruited 110 HNC patients. All participants were assessed for the severity of depression and anxiety symptoms, and the degrees of internalized stigma and its components, at each timepoint.

**Results:** MBSR significantly reduced the severity of depression and anxiety symptoms and degrees of internalized stigma and its components across timepoints (*T*_0_, *T*_1_, and *T*_2_). In contrast, no reduction in scores was observed in the TAU group. Furthermore, internalized stigma, SWA, and SSC partially mediated the relationship between MBSR and the severity of depression and anxiety symptoms. Sensitivity analyses confirmed that the changes in the severity of depression and anxiety symptoms and degrees of internalized stigma and its components according to intention-to-treat (ITT) analysis were similar to that of per-protocol (PP) and last observation carry forward (LOCF) analyses.

**Conclusion:** MBSR could be recommended as part of the treatment regimen for HNC patients.

**Trial Registration:** ClinicalTrials.gov identifier: NCT06991309

## 1. Introduction

Head and neck cancer (HNC) was the 7^th^ most prevalent cancer type globally in 2020, and by 2030, it is expected to rise at an alarming rate due to its causal relationship with human papillomavirus (HPV) infection [1, 2]. HNC patients are prone to facial disfigurement and various physical symptoms, such as loss of smell and taste, hearing difficulties, dyspnea, and challenges in swallowing, speaking, and social interaction [3]. These physical challenges often lead to emotional turmoil, including depression and anxiety.

The prevalence of depression and anxiety among HNC patients ranges from 20% to 40%, which is higher compared to other types of cancer [4]. Moreover, HNC complicated by depression and anxiety increases the risk of suicidal behavior. A systematic review and meta-analysis of HNC patients reported a pooled incidence of suicide at 161.16 per 100,000 individuals per year [3]. Depression and anxiety contribute to several negative outcomes for HNC patients. These conditions lower their quality of life [5]. Regarding survival outcomes, HNC patients diagnosed with depression both prior to and after their cancer diagnosis are more likely to die from cancer and other causes compared to those without depression [5]. Therefore, it is crucial to study depression, anxiety, and effective psychological interventions for HNC patients.

In addition, stigma is a significant factor closely related to depression and anxiety in cancer patients. Stigma is defined as a negative perception experienced by an individual due to their perceived differences from others. Internalized stigma, in particular, is strongly associated with depression and anxiety. It refers to irrational feelings, sentiments, and behaviors directed toward oneself, accompanied by a belief that one is a devalued member of society due to the label associated with their illness [6]. Stigma related to cancer has been linked not only to depression and anxiety but also to impaired physical health, psychological well-being, and social functioning, especially in HNC patients with facial disfigurement. These patients often experience devaluation of their social identity, leading to internalized stigma [7]. Internalized stigma implies to the phenomenon in which cancer patients adopt the negative societal perception about cancer and apply these perceptions to themselves, resulting in feelings of shame, self-blame, and sense of worthlessness. Research also shows that the degree of internalized stigma tends to increase over time after treatment completion in HNC patients [8]. Given its detrimental impact, internalized stigma warrants attention as a potential target for psychological interventions among cancer patients.

Mindfulness-based interventions (MBIs) are widely recognized as effective treatments for depression and anxiety in cancer patients. Mindfulness refers to the practice of maintaining awareness of the present moment, acknowledging and accepting thoughts, emotions, and behaviors as they arise, without judgment or avoidance [9]. Mindfulness-based stress reduction (MBSR) is a commonly practiced MBI that typically consists of 8 weekly group sessions. These sessions incorporate mindfulness strategies, meditation, and gentle yoga to address stress, pain, and various illnesses.

The benefits of MBSR can be explained through the mindfulness-to-meaning theory (MMT), which describes how mindfulness facilitates positive emotional regulation and promotes eudaimonic well-being—a state of meaningful and healthy living. According to MMT, MBSR helps individuals shift their attentional focus away from stress-related stimuli, maladaptive behaviors, and cognitive biases toward a metacognitive state of awareness, characterized by decentering. This shift allows for the acceptance of all experiences, whether positive or negative, in a nonjudgmental manner. As a result, individuals can reappraise stressors positively, experience greater positive affect, and find meaning in life [10]. This contributes to alleviation of stress and negative emotional sequelae (such as depression and anxiety) related to living with cancer and the adverse effects of its treatment. In addition, decentering followed by attainment of metacognitive state of awareness allow an MBSR practitioner to observe one's thought and feelings about the internalized stigma of living with cancer in a detached manner, ultimately leading to acceptance and acknowledgement of the internalized stigma in a nonjudgmental manner. Hence, MBSR reduces the degree of internalized stigma on living with cancer and the components of stigma such as sense of stigma (SS) and shame with appearance (SWA). This in turn ameliorate the speech and social concerns (SSCs) of cancer patients as SS and shame diminished. Moreover, MBSR through decentering and metacognitive awareness may improve self-compassion which facilitates acceptance of feelings of regret over past wrong-doing or goals which have not been accomplished before diagnosis of cancer [11–13].

Despite its proven effectiveness, data on the use of MBSR to treat depression and anxiety in HNC patients are limited. To date, no research has explored the effectiveness of MBSR in managing internalized stigma and its components among HNC patients. Given the high prevalence of depression, anxiety, and internalized stigma in this population, addressing this research gap is essential. Hence, this study aimed to investigate the effectiveness of MBSR in HNC patients by: (1) comparing the rate of change of the severity of depression and anxiety symptoms (primary outcomes) and the degrees of internalized stigma and its components (secondary outcomes) between MBSR and treatment-as-usual (TAU) control groups across three timepoints (*T*_0_ = baseline assessment, prior to intervention; *T*_1_ = postintervention, immediately after completion of intervention which was at 8 weeks after commence of intervention; *T*_2_ = follow-up assessment, 12 weeks after completion of intervention), and to determine the mechanism underlying the effectiveness of MBSR to alleviate depression and anxiety by: (2) assessing the mediation effect of decreasing the degree of internalized stigma and its components on the relationship between MBSR and the severity of depression and anxiety symptoms among HNC patients.

## 2. Methods

### 2.1. Study Design and Subject Recruitment

This multicenter, two-armed, parallel, and double-blind randomized controlled trial (RCT) was conducted between January 2022 and June 2023. The study was approved by the institutional review boards (IRBs) of two oncology referral centers located in the northern and central regions of Peninsular Malaysia. It adhered to the ethical principles outlined in the Helsinki Declaration of 1964 and its subsequent amendments. There was no changes to the trial design and methodology and the study was conducted according to the research protocol approved by the IRB.

The sample size was estimated using the sample size calculator developed by Lu et al. [14] for mixed linear models. Key parameters included a standard mean difference (SMD) of 0.6 (based on the SMD of a RCT on the effect of MBSR on cancer stigma among lung cancer and colorectal cancer patients [15, 16], as this is the only study which investigated the effect of MBSR on cancer patients), a type I error of 0.05, power of 0.8, a 1:1 group allocation ratio, three timepoints, and estimated attrition rates of 20% (from *T*_0_ to *T*_1_) and 20% (from *T*_1_ to *T*_2_). Based on these calculations, the estimated sample size was 54 participants per group.

The participants in this study was partly drawn from a RCT investigating the effects of MBSR onto positive psychological traits and experiential avoidance among HNC patients [17]. Potential participants were recruited from the pool of HNC patients registered at the two oncology referral centers. Recruitment efforts included posters displayed on notice boards within the centers and in areas surrounding the Oncology, Otorhinolaryngology, and Oral and Maxillofacial Surgery clinics, as well as inpatient wards. A research assistant, who was not involved in the study and not aware of its objectives, approached potential participants. All potential participants were screened for inclusion and exclusion criteria of the study. The inclusion criteria included: (1) those diagnosed with HNC confirmed by histopathological report and at any stage of cancer; (2) those aged 18 years old and above; (3) those who have completed surgery and started chemotherapy and/or radiotherapy; (4) literate in the Malay language. While the exclusion criteria were: (1) history of preexisting psychiatric illnesses (such as psychotic disorder, bipolar disorder, anxiety disorder, depressive disorder, neurodevelopmental disorder, and neurocognitive disorder). By excluding cancer patients with preexisting psychiatric illnesses, the findings of the study could be more precisely attributed to changes in the degree of depression, anxiety. and internalized stigma due to MBSR as preexisting psychiatric illnesses could be confounding factors which interfere with the effect of MBSR. For example systematic reviews and meta-analyses reported that MBSR exerted no or small to moderate beneficial effects on reduction of stress, depression, and anxiety across timepoints in studies which excluded participants with preexisting psychiatric illnesses compared with studies which included participants with preexisting psychiatric illnesses, whereby the latter revealed large effect of MBSR on reduction of stress, depression, and anxiety across timepoints [18]; (2) history of preexisting medical illnesses which may induce psychiatric symptoms (such as kidney and liver failure, ischemic heart disease, epilepsy, hyperthyroidism, hypothyroidism, Cushing's syndrome, Addison's disease, systemic lupus erythematosus, neurological disorder, rheumatoid arthritis); (3) history of illicit drug and alcohol use; (4) pregnancy; (5) suicidal tendency; (6) those with history of engaged in other psychotherapy; (7) those who are on psychotropic medications for depression and anxiety disorders within the last 4 months prior to enrollment in the study (4 months may be needed for total washout of psychotropic medication [19]; (8) those who are not physically and cognitively fit to participate in the study. Those who fulfilled all inclusion criteria without any exclusion criteria were invited to participate in the study and signed informed consent for study participation before they were enrolled in the study.

### 2.2. Randomization

Participants were then randomized into specific groups by stratified permuted block randomization which was carried out by a trained research assistant who is not involved in the study and not aware of the objectives of the study. Initially, participants were stratified according to gender (female and male) and stage of cancer (stage 1, 2, 3, and 4). Then, participants were randomized to MBSR intervention and TAU control groups in a ratio of 1 : 1, in which each participants received a computer-generated sequential number enclosed in an opaque envelope for allocation instruction to their designated group.

### 2.3. Intervention

Participants in the MBSR group received MBSR intervention, while those in the TAU control group participated in nontherapeutic management. Both groups attended 1-h weekly sessions for 6 weeks. Per-protocol (PP), participants who attended at least five sessions were considered to have achieved an adequate and acceptable level of attendance. Each session was conducted in groups of 10 participants.

#### 2.3.1. MBSR Group

Participants in the MBSR group attended 2.5 h of weekly sessions followed by 45 min of home assignments or practice over 8 weeks. The MBSR intervention was based on the format developed by Kabat-Zinn [20]. The sessions introduced participants to various mindfulness strategies, including mindfulness breathing, body scan, raisin exercise, mindfulness movement, 3-step breathing space, thought observation, metaphorical practice, practice of the vicious flower, clarifying life priorities and practice of kindness. Each participant was also provided with workbook and VCD of various meditation and mindfulness exercise for their daily practice of MBSR at home.

Cultural adaptation of the MBSR sessions was based on the diverse multiethnic background of Malaysia which include: (a) translation of the MBSR manual, workbooks, VCD, and materials into the Malay language which is the national language of Malaysia, (b) use of metaphors that represent the living environment and daily life in Malaysia, (c) conduct of yoga, mindful pose and body movements with respect to its Hindu and Buddhist origin as well as avoid movement or pose which has potential conflicting belief with Islam, and (d) scenarios for practice of mindfulness techniques and construction of metacognitive awareness were derived from the common day-to-day events in Malaysia. The details of each MBSR sessions are presented in Supporting Information 1: File 1.

#### 2.3.2. TAU Control Group

The participants in the control group will receive TAU in which routine oncology support such as appointment with oncologists or palliative care physicians, general practitioner for usual cancer care. The participants physical health and medications for their cancer treatment will be reviewed and treatment will be modified according to physical symptoms such as pain. Specific psychological interventions such as cognitive behavioral therapy, interpersonal therapy, MBIs, acceptance and commitment therapy, et cetera, will be avoided.

#### 2.3.3. Monitoring of Home Assignments

Participants conduct of home assignments was also monitored as they were required to report their conduct of home assignments through an online form (Google form) and reports of reasons of why participants missed intervention sessions and home assignments were also recorded through online form. There were several objective measures used to minimize reporting bias of the participants through the online platform which included: (1) participants were encouraged to openly discuss any difficulties or challenges to engage in the MBSR home assignments, (2) therapists actively address any barriers to the conduct of home assignments (such as lack of motivation, lack of time, and physical exhaustion) by suggesting possible solutions to be carried out by the participants at home, (3) regular checking by random home visits by the therapists as per agreement with participants to discuss on obstacles, effectiveness of suggested solutions, and support to engage in regular home assignments, (4) small incentive offered to participants who genuinely engage in home assignments, and (5) regular review of online entry by participants to monitor the online data and adjust the MBSR home assignments as necessary to optimize engagement of home assignments by the participants.

### 2.4. Treatment Fidelity

To ensure treatment fidelity, four therapists from each of the targeted center were selected to conduct the MBSR and nontherapeutic sessions in the MBSR and TAU control groups, respectively. These therapists were postgraduate psychology students with at least 2 years of experience conducting psychotherapy. Importantly, the therapists were not involved in the study and were unaware of its objectives.

Initially, all selected therapists participated in a 26-h session time which include eight classes of 2.5 h and an all-day class [21] conducted by an experienced psychiatrist and a clinical psychologist trained in MBSR and not involved in the study and were unaware of its objectives. Only after completing this training were they permitted to independently facilitate MBSR sessions for the MBSR group.

To further ensure fidelity, 15% of the video-audio recordings of each therapist's sessions were randomly selected from the beginning, middle, and end of the MBSR sessions. These recordings were assessed by the experienced psychiatrists and clinical psychologist using the MBIs: Teaching Assessment Criteria (MBI-TAC). Based on the RCT which evaluated the effect of MBSR, random selection of 15%–20% of the audiovisual recording of the MBSR sessions from each of the therapists was sufficient to assess the treatment fidelity among the therapists [22]. The inter-rater correlation coefficient for these assessments was calculated and found to be 0.75, indicating substantial agreement. It was suggested that a range of kappa findings from 0.61 to 0.80 is considered as substantial agreement [23]. Any issues or challenges encountered during the MBSR sessions were reported and discussed with the experienced psychiatrist to resolve them effectively.

### 2.5. Blinding

This study implemented double blinding. The researchers were blinded as subject recruitment, randomization, and data collection were conducted by research assistants who were not involved in the study and were unaware of its objectives. Data analysis, conducted after the completion of data collection, was carried out by statisticians who were also uninvolved in the study and blinded to its objectives. The statisticians followed a preplanned statistical manual as guidance for the analysis. Once data collection and analysis were completed, the researchers were unblinded to proceed with the interpretation and reporting of the findings. Additionally, the MBSR and nontherapeutic sessions for the MBSR and TAU control groups were conducted by psychotherapists who were not involved in the study and were unaware of its objectives.

We were unable to completely blind the participants in this study but their randomization and allocation to their respective group were carried out by research assistants who were not involved in the study and were unaware of its objectives with the allocation sequence enclosed in an opaque envelope to conceal their group allocation. Participants in the TAU control group were offered the MBSR intervention only after completing the study.

### 2.6. Withdrawal and Exit Criteria

The participants could withdraw at any point of the study. In addition, participants were allowed to exit from the study if: (1) they developed adverse effects related or unrelated to the intervention, (2) they developed suicidal tendency while they were still participating in the study, and (3) they received other treatment while they were still participating in the study.

### 2.7. Data Collection

All the participants were administered: (1) sociodemographic and clinical characteristics questionnaire, (2) the Malay version of the Hospital Anxiety and Depression Scale (HADS) to assess the degree of severity of their anxiety and depression symptoms, and (3) the Malay version of the Shame and Stigma Scale (SSS) to measure the degree of internalized stigma during baseline assessment (*T*_0_; prior to intervention). Then, the HADS and SSS were re-administered to all the participants at postintervention (*T*_1_; at 8 weeks after intervention began or immediately after completion of the intervention) and at follow-up (*T*_2_; at 12 weeks after completion of the intervention). To ensure treatment adherence and compliance to assessment sessions, phone calls were made 3 days prior to remind participants of their next intervention and assessment appointments. Those who did not attend their assessment appointment were also reminded through telephone call. This study was conducted according to the CONSORT guidelines and a copy of the CONSORT checklist is presented as Supporting Information 2: File 2.

### 2.8. Measures

#### 2.8.1. Primary Outcomes (Depression and Anxiety)

##### 2.8.1.1. HADS

HADS is a self-administered tool used to assess the degree of severity of depression and anxiety symptoms among the participants in this study. HADS consists of two subscales (depression and anxiety), in which each subscale consists of seven items. Each item is scored in a Likert scale ranging from 0 to 3, whereby the higher total subscale score indicates greater severity of depression and anxiety symptoms. The cut-off score for possibility of having depression is 8/21 for the HADS depression subscale and possibility for occurrence of anxiety disorder is 8/21 for the HADS anxiety subscale [24]. The HADS was translated and validated in the Malay language, in which the Malay version of HADS exhibited good internal consistency with Cronbach's *α* ranging from 0.73 to 0.87 for its subscales [25].

#### 2.8.2. Secondary Outcomes (Internalized Stigma)

##### 2.8.2.1. SSS

The SSS is a self-administered instrument employed to measure the degree of internalized stigma of living with cancer among the participants in this study. It consists of 20 items and made up of four domains (SWA, speech and social concerns, sense of stigma, and regret). Each item is scored in a Likert scale ranging from 0 (never) to 4 (all the time). Hence, the higher the score, the greater is the degree of internalized stigma. The SSS exhibits good psychometric properties with an excellent internal consistency (Cronbach's *α* = 0.94) [26]. It was translated and validated in the Malay language among the cancer patients population in Malaysia and demonstrated good internal consistency with Cronbach's *α* of 0.88 [27].

#### 2.8.3. Sociodemographic and Clinical Characteristics

Sociodemographic characteristics recorded from the participants included age, gender, ethnicity, and marital status. While the clinical characteristics recorded include types of HNC, stage of cancer, and duration since diagnosis. The response options for age were coded as 18–45 years and 46 years and above. The responses for gender were male and female. The response options for ethnicity were Malays and non-Malays. The responses for marital status were married and single/widow/widower/divorcee. The response options for types of HNC were nasopharyngeal carcinoma and other types of HNC. The response options for time since diagnosis were new case and 1–6 months. The response options for stage of cancer were stage 1and 2 and stage 3 and 4.

### 2.9. Statistical Analysis

Data analysis was carried out with Statistical Package for Social Sciences version 28 (SPSS 28). Descriptive statistics was computed for sociodemographic and clinical characteristics for both the MBSR and TAU control groups. Sociodemographic and clinical characteristics were documented in frequency and percentage. The differences in the sociodemographic and clinical characteristics between the MBSR and TAU control groups were assessed using Pearson's chi square test and Fisher exact test (the latter test if any cell had less than five subjects). In addition, the differences in the sociodemographic and clinical characteristics of participants who drop-out before completion of the study between the MBSR and TAU groups were also compared using Fisher exact test.

To achieve objective (1), mixed linear model was employed to evaluate the rate of change of the HADS depression and anxiety (primary outcomes), the SSS and its domain scores (secondary outcomes) across the three timepoints (*T*_0_, *T*_1_, and *T*_2_) while controlled for confounding factors such as age, gender, types of HNC, and time since diagnosis [28–30]. Mixed linear model is preferred over mixed ANOVA as it enabled data from those who had not completed all timepoints to be utilized, hence enable intention-to-treat (ITT) analysis to be carried out. From the mixed linear model, those with significant interaction between time and group were subjected to: (1) post hoc analysis to examine the differences in the HADS depression and anxiety, the SSS and its domain scores between the MBSR and TAU groups at each timepoints (between subjects difference) and (2) post hoc analysis to evaluate the differences in the HADS depression and anxiety, the SSS and its domain scores across the three timepoints in each of the group (MBSR and TAU) (within subject differences). The normality of the residuals (the differences in observed and predicted values) were examined using Q–Q plot of the residuals and if non-normal distribution of residuals confirmed, bootstrapping with 5000 replications was performed. The standardized mean difference (effect size) was reported as Cohen's *d*:

  $$\begin{array}{c} d={\text{ Mean}}_{1}-{\text{ Mean}}_{2}/\text{pooled SD}, \end{array}$$

  $$\begin{array}{c} \text{pooled SD}=\surd {{\text{ SD}}_{1}}^{2}+{{\text{ SD}}_{2}}^{2}/2, \end{array}$$

where SD = standard deviation.

Cohen's *d* of 0.2 was considered small effect size, while 0.5 was designated as moderate effect size and 0.8 was denoted as large effect size.

Then, sensitivity analysis was performed by comparing the mixed linear regression findings, including the posthoc between subject and within subject analyses for the HADS depression and anxiety, the SSS and its domain scores assessed between ITT, PP and last observation carry forward (LOCF). According to the PP analysis, those did not attend for at least five intervention sessions in the MBSR and TAU groups will be excluded.

In order to achieve objective (2), initially the Pearson's correlation coefficient between internalized stigma, severity of depression and anxiety symptoms were computed. Then, the mediation effect of internalized stigma (mediator) on the relationship between MBSR (independent variable) and the mean difference of the HADS depression subscale and anxiety subscale scores between *T*_0_ and *T*_2_ (dependent variable) were evaluated with PROCESS macro-version 4.2 developed by Andrew F. Hayes. The direct and indirect effect of MBSR on the severity of depression and anxiety symptoms were computed with bootstrapping with 5000 replications. If the direct effect of MBSR on severity of depression and anxiety symptoms were less than that of total effect, while the indirect effect of internalized stigma on the relationship between MBSR and severity of depression and anxiety symptoms were significant, then internalized stigma exert mediation effect on the relationship between MBSR and severity of depression and anxiety symptoms. Statistical significance was set at *p*  < 0.05 and was two-sided.

## 3. Results

### 3.1. Participants

**Figure 1:**
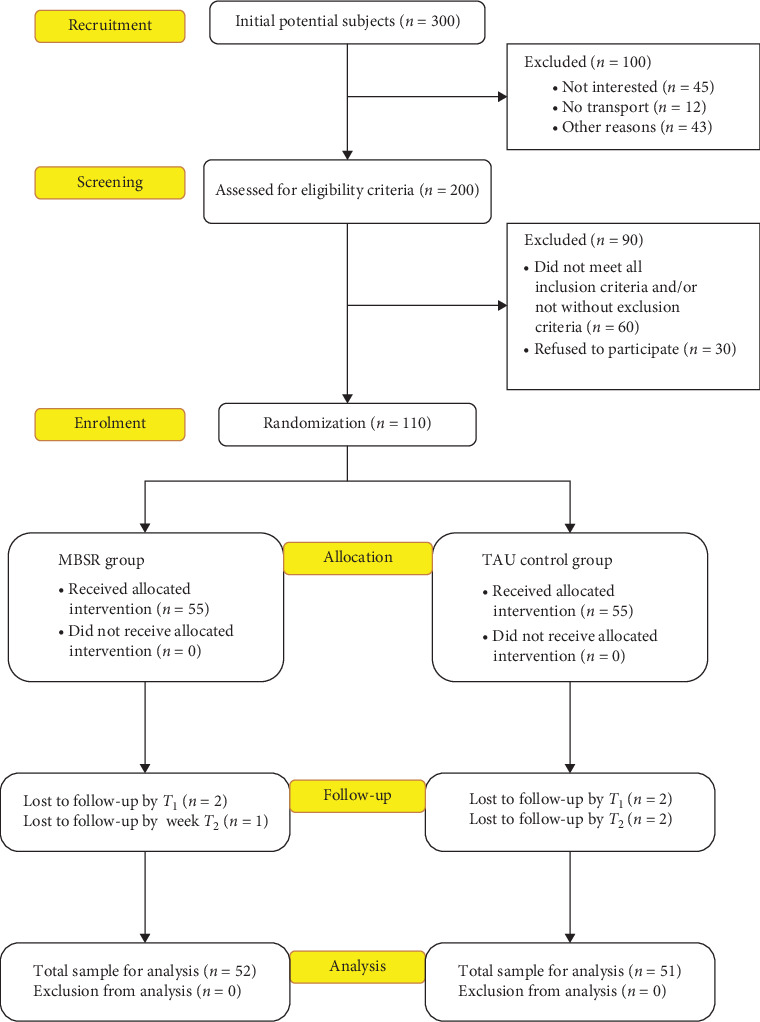
The chronology of subject recruitment in this study.

The chronology of subject recruitment in this study is illustrated in Figure 1. Initially, 300 subjects from the two targeted centers were approached, but 100 subjects were excluded due to lack of interest to participate in the study (45 subjects), lack of transport to travel to the centers (12 subjects), and citing other reasons (43 subjects). Then, 200 subjects were screened for eligibility to participate in the study but another 90 subjects were excluded due to failure to meet the eligibility criteria (60 subjects) and refuse to participate when offered (30 subjects). Hence, a total of 110 subjects were enrolled in the study and randomized into the MBSR (*n* = 55) and TAU control groups (*n* = 55). Then, during the postintervention assessment (*T*_1_), two participants from the MBSR group did not turn up for assessment, while 53 participants in the MBSR group completed all assessments. Similarly, two participants from the TAU group also did not turn up for assessment, while 53 participants in the TAU group completed all assessments. Finally, during the follow-up assessment (*T*_2_), one more participant from the MBSR group was absent for assessment, while two more participants from the TAU group was absent for assessment. Hence, a total of 52 participants completed assessments in all three timepoints in the MBSR group (attrition rate = 5.5%), while a total of 51 participants completed assessments in all timepoints in the TAU group (attrition rate = 7.3%). The study was not associated with occurrence of any adverse effects or harm.

Regarding the PP analysis, a total of four participants from the MBSR group attended less than five intervention sessions, in which two participants only attended two sessions while another two participants attended only four sessions. While in the TAU group, a total of five participants failed to attend for at least five intervention sessions, whereby two participants attended only two sessions, one attended only three sessions and two more attended only four sessions.

The sociodemographic and clinical characteristics of all the participants are summarized in Table 1. There was no significant differences in the sociodemographic and clinical characteristics between the MBSR and TAU control groups. In addition, there was no differences in age, gender, ethnicity, marital status, types of HNC, stage of cancer, and duration since diagnosis distributions between the participants in the MBSR and TAU control groups who drop-out from the study before its completion (*p*  > 0.05).

### 3.2. Rate of Change in the HADS Depression and Anxiety Subscale Scores (Primary Outcomes) Between the MBSR and TAU Control Group Across the Three Timepoints (*T*_0_, *T*_1_, and *T*_2_) While Controlling for Confounding Factors According to the ITT Analysis

The main effects of between groups (between subject factor), timepoints (within subject factor) and the interaction between groups and timepoints of the HADS and the total SSS scores according to ITT analysis are summarized in Table 2. Based on the linear mixed model, the main effect of the interaction between group and time for the HADS anxiety score (*F* [2, 208] = 32.591, *p*  < 0.001) and the main effect of time (*F* [2, 208] = 3.956, *p* = 0.044) were significant after controlling for confounding factors (such as age, gender, types of HNC, and time since diagnosis). However, the main effect of between groups was not significant (*p* = 0.338). As for HADS depression score, the main effect of interaction between group and time (*F* [2, 206] = 31.575, *p*  < 0.001), the main effect of time (*F* [2, 206] = 7.986, *p*  < 0.001) and the main effect of between groups (*F* [1, 112] = 13.727, *p*  < 0.001) were significant after controlling for confounding factors (such as age, gender, types of HNC, and time since diagnosis).

The post hoc assessment for HADS depression and anxiety subscale and total SSS scores between the MBSR and TAU groups in each timepoints (*T*_0_, *T*_1_, and *T*_2_; between subjects analysis) following the ITT analysis are summarized in Table 3. While the post hoc assessment for HADS depression and anxiety subscale and total SSS scores across the three timepoints (*T*_0_, *T*_1_, and *T*_2_) within the MBSR and the TAU groups (within subject analysis) following ITT analysis are presented in Table 4. According to the post hoc between subjects assessment (Table 3), at baseline (*T*_0_), there was no difference in the HADS (depression) score between MBSR and TAU groups (*p* = 0.424). Then at postintervention (*T*_1_), the HADS (depression) score in the MBSR group declined and was significantly lower than that in the TAU group (adjusted mean difference = −3.176, 95% CI = −4.620 to −1.732, *p*  < 0.001) with large effect size (standardized mean difference [SMD] = −0.926). At follow-up (*T*_2_), the HADS (depression) score in the MBSR group further declined and resulting in significantly lower score compared with the TAU group (adjusted mean difference = −4.588, 95% CI = −6.048 to −3.127, *p*  < 0.001) with an even larger effect size (SMD = −1.263). The post hoc within subject assessment (Table 4) documented significant decline in the HADS (depression) scores in the MBSR group from baseline (*T*_0_) to postintervention (*T*_1_; *p*  < 0.001) and from postintervention (*T*_1_) to follow-up (*T*_2_; *p* = 0.045), while the HADS score in the TAU group did not significantly changed from baseline (*T*_0_) to postintervention (*T*_1_; *p* = 0.061) and from postintervention (*T*_1_) to follow-up (*T*_2_) (*p* = 0.644).

As for the HADS anxiety subscale score, according to the post hoc between subjects assessment (Table 3), at baseline (*T*_0_), the HADS (anxiety) score of the MBSR group was significantly higher than that of the TAU group (adjusted mean difference = 2.255, 95% CI = 0.962 to 3.548, *p*  < 0.001). However, as the HADS (anxiety) score of the MBSR group decreased at postintervention (*T*_1_), while the HADS (anxiety) score of the TAU group increased, there was no significant difference documented in the HADS (anxiety score) between the MBSR and TAU groups (*p* = 0.055). As the HADS (anxiety) score in the MBSR group continued to decline further at follow-up (*T*_2_), the HADS (anxiety) score of the MBSR group was significantly lower than that of the TAU group (adjusted mean difference = −2.651, 95% CI = −3.971 to −1.331, *p*  < 0.001) with a medium effect size (SMD = −0.776). While in the post hoc within subject assessment (Table 4), the HADS (anxiety) scores in the MBSR group significantly decreased from baseline (*T*_0_) to postintervention (*T*_1_; *p*  < 0.001) and from postintervention (*T*_1_) to follow-up (*T*_2_; *p* = 0.011), while the HADS (Anxiety) scores in the TAU group significantly increased from baseline (*T*_0_) to postintervention (*T*_1_; *p*  < 0.001), but did not significantly changed from postintervention (*T*_1_) to follow-up (*T*_2_; *p* = 1.000).

### 3.3. Rate of Change in the SSS and Its Domain Scores (Secondary Outcomes) Between the MBSR and TAU Control Group Across the Three Timepoints (*T*_0_, *T*_1_, and *T*_2_) While Controlling for Confounding Factors According to the ITT Analysis

The linear mixed model for the total SSS score revealed that the main effect for the interaction between group and time (*F* [2, 212] = 12.744, *p*  < 0.001), the main effect of timepoints (*F* [2, 212] = 5.791, *p* = 0.004) and the main effect of between groups (*F* [1, 115] = 22.286, *p*  < 0.001) were significant after controlling for confounding factors (such as age, gender, types of cancer, and time since diagnosis; Table 2).

The main effects of between groups (between subject factor), timepoints (within subject factor), and the interaction between groups and timepoints of the SSS domain scores according to ITT analysis are summarized in Table 5. In the context of the SSS domains, for SWA, SSC, and SS; the main effect for the interaction between group and time, the main effect of time, and the effect of between groups were significant (*p*  < 0.05) after controlling for confounding factors (such as age, gender, types of cancer, and time since diagnosis). However, as for the regret (R) domain, the main effect of between groups and the main effect of the interaction between group and time were significant (*p*  < 0.05) after controlling for confounding factors (such as age, gender, types of cancer, and time since diagnosis), but not the main effects of time which was not significant (*p* = 0.217).

According to the post hoc between subjects assessment (Table 3), at baseline (*T*_0_), the total SSS score of the MBSR group was significantly lower than that of the TAU group (adjusted mean difference = −7.820, 95% CI = −13.785 to −1.854, *p* = 0.011) with medium effect size (SMD = −0.552). Then at postintervention (*T*_1_), the total SSS score in the MBSR group was further decline, resulting in increase in the adjusted mean difference between the MBSR and TAU groups (adjusted mean difference = −13.450, 95% CI = −19.445 to −7.456, *p*  < 0.001) with large effect size (SMD = −0.864). At follow-up (*T*_2_), the total SSS score in the MBSR group continued to further decline and resulting in a further increase in the mean difference between the MBSR and TAU groups (adjusted mean difference = −18.990, 95% CI = −25.029 to −12.951, *p*  < 0.001) with an even larger effect size (SMD = −1.201). The post hoc within subject assessment (Table 4) documented significant decline in the total SSS scores in the MBSR group from baseline (*T*_0_) to post intervention (*T*_1_; *p*  < 0.001) and from post intervention (*T*_1_) to follow-up (*T*_2_; *p*  < 0.001), while the total SSS score in the TAU group remained unchanged from baseline (*T*_0_) to postintervention (*T*_1_; *p* = 1.000) and from postintervention (*T*_1_) to follow-up (*T*_2_; *p* = 1.000).

The post hoc assessment for the SSS domain scores between the MBSR and TAU groups in each timepoints (*T*_0_, *T*_1_, and *T*_2_; between subjects analysis) following the ITT analysis are summarized in Table 6. While the post hoc assessment for the SSS domain scores across the three timepoints (*T*_0_, *T*_1_, and *T*_2_) within the MBSR and the TAU groups (within subject analysis) following ITT analysis are presented in Table 7. In the context of the SSC domain, the post hoc between subject analysis shown that at baseline (*T*_0_), the SSC score of the MBSR group was significantly lower than that of the TAU group (*p*  < 0.001) with medium effect size (SMD = −0.789). At postintervention (*T*_1_), the scores decline in the MBSR group resulting in the score of MBSR group to be further lowered compared with that of the TAU group (*p*  < 0.001) with further increase in effect size (SMD = −0.934). Then at follow-up (*T*_2_), the SSC score further decline contributing to further lowered compared with that of the TAU group (*p*  < 0.001) with the largest effect size compared with other timepoints (SMD = −1.205) (Table 6). The post hoc within subject assessment (Table 7) in the MBSR group demonstrated a decrease in the SSC score from baseline (*T*_0_) to postintervention (*T*_1_) but was not statistically significant (*p* = 0.057). While the SSC score significantly decrease from postintervention (*T*_1_) to follow-up (*T*_2_) in the MBSR group (*p* = 0.021). Whilst the SSC score in the TAU group did not significantly changed from baseline (*T*_0_) to postintervention (*T*_1_) (*p* = 1.000) and from postintervention (*T*_1_) to follow-up (*T*_2_) (*p* = 1.000).

With regard to the SWA and the SS domains, post hoc between subject analysis indicated that there was no difference in the scores between MBSR and TAU groups at baseline (*T*_0_; *p*  > 0.05), but decline in the scores of the two domains in the MBSR group at postintervention (*T*_1_) resulting in a significantly lower SWA and SS scores compared with that of the TAU group (*p*  < 0.001). Further decline in the SWA and SS scores in the MBSR group at follow-up (*T*_2_) contributed to further lowering of scores in the MBSR group compared with that of the TAU group (*p*  < 0.001) (Table 6). The post hoc within subject assessment (Table 7) documented significant decline in the SWA scores in the MBSR group from baseline (*T*_0_) to post intervention (*T*_1_; *p*  < 0.001). While there was further decline in the SWA and SS scores from postintervention (*T*_1_) to follow-up (*T*_2_), the decline in scores were not statistically significant (*p*  > 0.05). Whilst the SWA and SS scores in the TAU group did not significantly changed from baseline (*T*_0_) to postintervention (*T*_1_) and from postintervention (*T*_1_) to follow-up (*T*_2_; *p*  > 0.05).

The post hoc between subject analysis shown that at baseline (*T*_0_), the regret score of the MBSR group was significantly lower than that of the TAU group (*p*  < 0.001) with medium effect size (SMD = −0.700). At postintervention (*T*_1_), the regret score remained unchanged in the MBSR group, whereby there was no increase in the effect size compared with *T*_0_ (SMD = −0.618). Then at follow-up (*T*_2_), the regret score decline contributing to further lower score in the MBSR group compared with that of the TAU group (*p*  < 0.001) with a larger effect size (SMD = −1.026) (Table 6). The post-hoc within subject assessment (Table 7) in the MBSR group demonstrated no significant change in the regret score from baseline (*T*_0_) to postintervention (*T*_1_; *p* = 1.000). While the regret score significantly decrease from postintervention (*T*_1_) to follow-up (*T*_2_) in the MBSR group (*p* = 0.037). Whilst the regret score in the TAU group did not significantly changed from baseline (*T*_0_) to postintervention (*T*_1_; *p* = 0.729) and from postintervention (*T*_1_) to follow-up (*T*_2_; *p* = 1.000).

### 3.4. Sensitivity Analyses

The main effects of between groups (between subject factor), timepoints (within subject factor) and the interaction between groups and timepoints of the HADS and the total SSS scores according to PP and LOCF analyses are summarized in Table 2. While the main effects of between groups (between subject factor), timepoints (within subject factor) and the interaction between groups and timepoints of the SSS domain scores according to PP and LOCF analyses are summarized in Table 5. Based on the linear mixed models according to the PP and LOCF analyses, for HADS (depression) and total SSS scores, the main effect of interaction between group and time, the main effect of time, and the main effect of between groups were significant (*p*  < 0.05) after controlling for confounding factors (such as age, gender, types of HNC, and time since diagnosis). While for HADS (anxiety) score, the main effect of the interaction between group and time, and the main effect of time were significant (*p*  < 0.05) after controlling for confounding factors (such as age, gender, types of HNC, and time since diagnosis), whilst the main effect of group was not significant (*p*  > 0.05) (Table 2).

While for the mixed linear models following the PP and LOCF analyses, the main effect for the interaction between group and time, the main effect of time, and the main effect of group were significant (*p*  < 0.05) for the SWA, SSC, and SS scores after controlling for confounding factors (such as age, gender, types of cancer, and time since diagnosis). On the other hand, as for regret (R), the main effect for the interaction between group and time, and the main effect of group were significant (*p*  < 0.05) after controlling for confounding factors (such as age, gender, types of cancer, and time since diagnosis), but not the main effect of time (*p*  > 0.05) (Table 5). Hence, when the mixed linear model findings of the HADS anxiety and depression subscale scores (primary outcomes), and total SSS and the SSS domain scores (secondary outcome) were compared, there were no differences between ITT, PP. and LOCF analyses.

Then, the post hoc assessment for HADS depression and anxiety subscale and total SSS scores between the MBSR and TAU groups in each timepoints (*T*_0_, *T*_1_, and *T*_2_; between subjects analysis) following PP and LOCF analyses are summarized in Supporting Information 3: Table S1. While the post hoc assessment for HADS depression and anxiety subscale and total SSS scores across the three timepoints (*T*_0_, *T*_1_, and *T*_2_) within the MBSR and the TAU groups (within subject analysis) following PP and LOCF analyses are presented in Supporting Information 4: Table S2. The post hoc assessment for the SSS domain scores between the MBSR and TAU groups in each timepoints (*T*_0_, *T*_1_, and *T*_2_; between subjects analysis) following the PP and LOCF analyses are summarized in Supporting Information 5: Table S3. While the post hoc assessment for the SSS domain scores across the three timepoints (*T*_0_, *T*_1_, and *T*_2_) within the MBSR and the TAU groups (within subject analysis) following PP and LOCF analyses are presented in Supporting Information 6: Table S4.

Based on the post hoc between subject comparison according to the PP and LOCF analyses, at baseline (*T*_0_), there was no difference in the HADS (depression) score between MBSR and TAU groups. As the HADS (depression) score reduced in the MBSR group at postintervention (*T*_1_) and follow-up (*T*_2_), the score in MBSR group was significantly lower than that of the TAU group (Supporting Information 3: Table S1). Based on the post hoc within subject comparison according to the PP and LOCF analyses, the HADS (depression) score significantly declined in the MBSR group from baseline (*T*_0_) to postintervention (*T*_1_) and from postintervention (*T*_1_) to follow-up (*T*_2_), while HADS (depression) score in the TAU group was significantly increased from baseline (*T*_0_) to postintervention (*T*_1_) and no significant change from postintervention (*T*_1_) to follow-up (*T*_2_) (Supporting Information 5: Table S3).

While based on the post hoc between subject comparison according to the PP and LOCF analyses, the HADS (anxiety) score at baseline (*T*_0_) was significantly higher in the MBSR than that of the TAU group. As the HADS (anxiety) score of the MBSR group declined, there was no significant difference in the score between MBSR and TAU group at postintervention (*T*_1_). As the HADS (anxiety) score further decreased in the MBSR group, the score in the MBSR group was significantly lower than that of the TAU group at follow-up (*T*_2_) (Supporting Information 3: Table S1). Based on the post hoc within subject comparison according to the PP and LOCF analyses, the HADS (anxiety) scores significantly decline in the MBSR group from baseline (*T*_0_) to postintervention (*T*_1_) and from postintervention (*T*_1_) to follow-up (*T*_2_), while HADS (depression) score in the TAU group was significantly increased from baseline (*T*_0_) to postintervention (*T*_1_), but remained unchanged from postintervention (*T*_1_) to follow-up (*T*_2_) (Supporting Information 5: Table S3). Hence, when the posthoc between subject and within subject comparison of the HADS anxiety and depression subscale scores (primary outcomes) were compared, there were no differences between ITT, PP, and LOCF analyses.

In the context of the total SSS score, the posthoc between subject comparison according to the PP and LOCF analyses revealed that the total SSS scores were significantly lower in the MBSR group compared with that of the TAU group at baseline (*T*_0_), postintervention (*T*_1_), and follow-up (*T*_2_) (Supporting Information 3: Table S1). Based on the post hoc within subject comparison according to the PP and LOCF analyses, the total SSS scores significantly declined in the MBSR group from baseline (*T*_0_) to postintervention (*T*_1_) and from postintervention (*T*_1_) to follow-up (*T*_2_). While the total SSS scores remained unchanged in the TAU group from baseline (*T*_0_) to postintervention (*T*_1_) and from postintervention (*T*_1_) to follow-up (*T*_2_) (Supporting Information 3: Table S3).

In term of the SSS domain scores, the post hoc between subject comparison according to the PP and LOCF analyses revealed that there were no difference in the SWA and SS scores between MBSR and TAU groups at baseline (*T*_0_). As the SWA and SS scores decreased in the MBSR group at postintervention (*T*_1_) and follow-up (*T*_2_), the scores of the MBSR group were significantly lower than that of the TAU group at *T*_1_ and *T*_2_ (Supporting Information 4: Table S2). Based on the post hoc within subject comparison according to the PP and LOCF analyses, the SWA score was significantly declined in the MBSR group from baseline (*T*_0_) to postintervention (*T*_1_), but remained unchanged from postintervention (*T*_1_) to follow-up (*T*_2_). Contrastingly, the SS score was significantly declined in the MBSR group from baseline (*T*_0_) to postintervention (*T*_1_) and from postintervention (*T*_1_) to follow-up (*T*_2_). While the SWA and SS scores exhibited no significant changes in the TAU group from baseline (*T*_0_) to postintervention (*T*_1_) and from postintervention (*T*_1_) to follow-up (*T*_2_) (Supporting Information 6: Table S4). Based on the post hoc between subject comparison according to the PP and LOCF analyses, the SSC and regret scores in the MBSR group were significantly lower than that of the TAU group at baseline (*T*_0_), postintervention (*T*_1_) and follow-up (*T*_2_) (Supporting Information 4: Table S2). Based on the post hoc within subject comparison according to the PP and LOCF analyses, the SSC and regret scores in the MBSR group did not exhibit significant difference compared with that of the TAU group from baseline (*T*_0_) to postintervention (*T*_1_), but the scores in the MBSR group significantly decreased from postintervention (*T*_1_) to follow-up (*T*_2_). Contrastingly, there were no significant differences in the SSC and regret scores in the TAU group from baseline (*T*_0_) to postintervention (*T*_1_) and from postintervention (*T*_1_) to follow-up (*T*_2_) (Supporting Information 6: Table S4). Hence, when the post hoc between subject and within subject comparison of the total SSS and its domain scores (secondary outcomes) were compared, there were no differences between ITT, PP, and LOCF analyses.

### 3.5. Mediation Analyses

**Figure 2:**
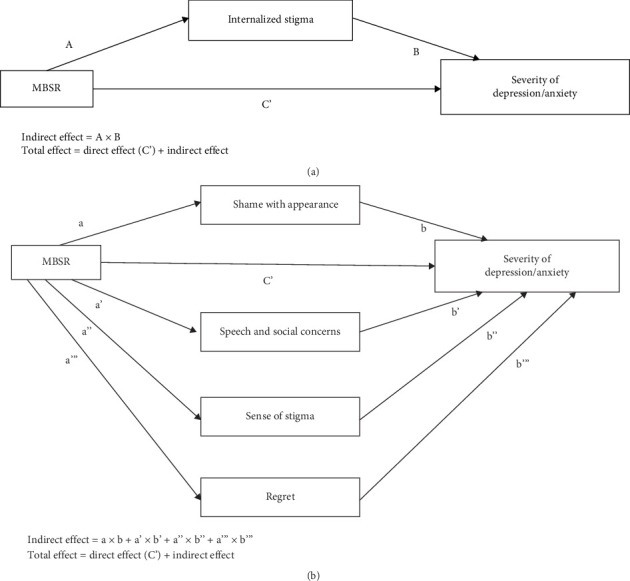
Diagrammatic presentation of the mediating effect of internalized stigma and its domains on the relationship between MBSR and the severity of depression and anxiety symptoms. Diagram (A) (internalized stigma as mediating factor): path A = the effect of MBSR on internalized stigma, path B = the effect of internalized stigma on the severity of depression/anxiety symptoms, and path C' = the direct effect of MBSR on the severity of depression/anxiety symptoms. Total indirect effect of internalized stigma = A × B. Total effect = C' + total indirect effect. Diagram (B) (domains of internalized stigma as mediating factors): path a = the effect of MBSR on shame with appearance, path b = the effect of shame with appearance on the severity of depression/anxiety symptoms, path a' = the effect of MBSR on speech and social concerns, path b' = the effect of speech and social concerns on the severity of depression/anxiety symptoms, path a” = the effect of MBSR on sense of stigma, path b” = the effect of sense of stigma on the severity of depression/anxiety symptoms, path a”' = the effect of MBSR on regret, and path b”' = the effect of regret on the severity of depression/anxiety symptoms. Total indirect effect of mediating factors = (a × b) + (a' × b') + (a” × b”) + (a”' × b”'). Total effect = C' + total indirect effect.

**Figure 3:**
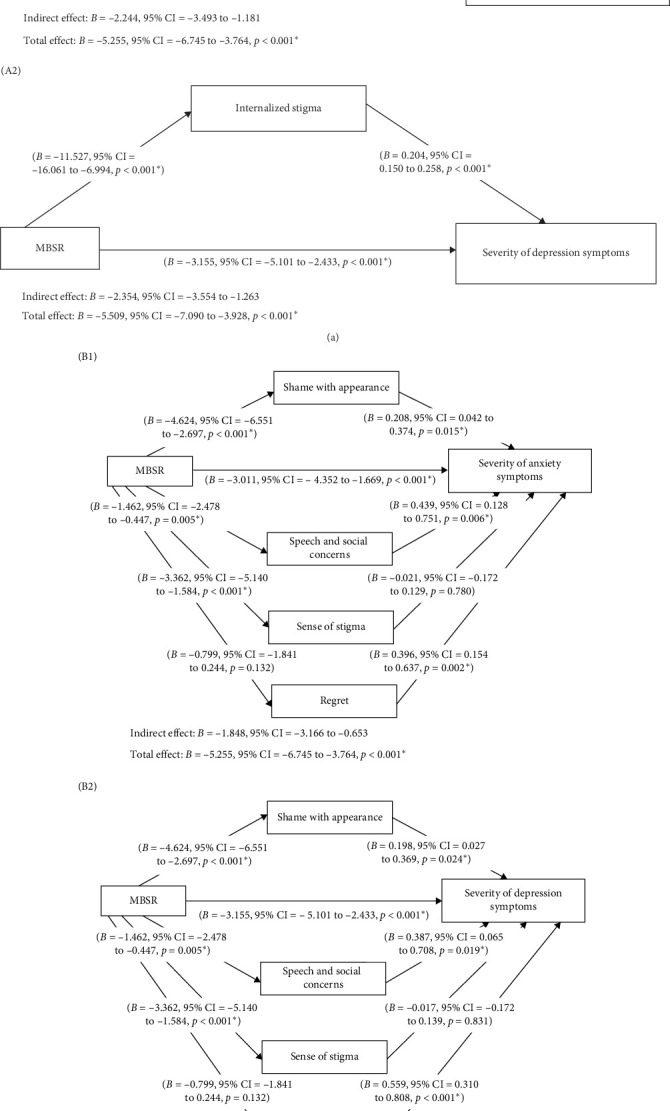
Mediating effect of internalized stigma and its domains on the relationship between MBSR and the severity of depression/anxiety symptoms among the head and neck cancer participants in this study. Diagram (A): MBSR significantly reduced the degree of internalized stigma, while internalized stigma significantly increase the severity of anxiety/depression symptoms. However, the direct effect of MBSR on the severity of anxiety/depression symptoms were less than that of total effect on reducing the severity of anxiety/depression symptoms. The total indirect effect of decreasing degree of internalized stigma significantly reduced the severity of anxiety/depression symptoms, indicating that internalized stigma exerted a partial mediation onto the relationship between MBSR and severity of anxiety/depression symptoms. Diagram (B): MBSR significantly reduced the degrees of shame with appearance, speech and social concerns, and sense of stigma, while shame with appearance, speech and social concerns, and regret significantly increase the severity of anxiety/depression symptoms. However, the direct effect of MBSR on the severity of anxiety/depression symptoms were less than that of total effect on reducing the severity of anxiety/depression symptoms. The total indirect effect of decreasing degrees of shame with appearance, speech and social concerns, sense of stigma, and regret significantly reduced the severity of anxiety/depression symptoms, but only the indirect effect of shame of appearance and speech and social concerns significantly decrease the severity of anxiety symptoms and only the indirect effect of speech and social concerns significantly decrease the severity of depression symptoms. Hence, shame of appearance and speech and social concerns exerted a partial mediation effect on the relationship between MBSR and anxiety symptoms, and speech and social concerns exerted a partial mediation effect on the relationship between MBSR and depression symptoms.

The mediation effect of internalized stigma and its domains on the relationship between MBSR and the severity of depression and anxiety symptoms among the HNC participants in this study are summarized in Tables 8 and 9 and Figures 2 and 3. In the context of internalized stigma as a mediating factor on the relationship between MBSR and severity of anxiety symptoms, MBSR exerted a significant effect on internalized stigma (*F* [1,108] = 25.400, *R*^2^ = 0.190, *p*  < 0.001), in which MBSR significantly decrease the degree of internalized stigma (*B* = −11.527, 95% CI = −16.061 to 6.994, *p*  < 0.001). MBSR and internalized stigma also exerted a significant effect on the severity of anxiety symptoms (*F* [2,107] = 66.105, *R*^2^ = 0.553, *p*  < 0.001), in which internalized stigma significantly increase severity of anxiety symptoms (*B* = 0.195, 95% CI = 0.144 to 0.245, *p*  < 0.001). The direct effect of MBSR on the severity of anxiety symptoms (*B* = −3.011, 95% CI = −4.352 to −1.669, *p*  < 0.001) was less than that of the total effect (*B* = −5.255, 95% CI = −6.745 to 3.764, *p*  < 0.001), while the total indirect effect of internalized stigma on the relationship between MBSR and the severity of anxiety symptoms was significant (*B* = −2.244, 95% CI = −3.493 to −1.181). In conclusion, the findings indicated that internalized stigma exerted a partial mediation effect onto the relationship between MBSR and the severity of anxiety symptoms among the HNC participants in this study. MBSR significantly decrease the severity of anxiety symptoms partially via declining degree of internalized stigma.

Similarly, regarding internalized stigma as a mediating factor on the relationship between MBSR and severity of depression symptoms, MBSR exerted a significant effect on internalized stigma (*F* [1,108] = 25.400, *R*^2^ = 0.190, *p*  < 0.001), in which MBSR significantly decrease the degree of internalized stigma (*B* = −11.527, 95% CI = −16.061 to 6.994, *p*  < 0.001). MBSR and internalized stigma also exerted a significant effect on the severity of depression symptoms (*F* [2,107] = 63.897, *R*^2^ = 0.544, *p*  < 0.001), in which internalized stigma significantly increase severity of depression symptoms (*B* = 0.204, 95% CI = 0.150 to 0.258, *p*  < 0.001). The direct effect of MBSR on the severity of anxiety symptoms (*B* = −3.155, 95% CI = −5.101 to 2.433, *p*  < 0.001) was less than that of the total effect (*B* = −5.509, 95% CI = −7.090 to 3.928, *p*  < 0.001), while the total indirect effect of internalized stigma on the relationship between MBSR and the severity of depression symptoms was significant (*B* = −2.354, 95% CI = −3.554 to 1.263). Hence, the findings indicated that internalized stigma exerted a partial mediation effect onto the relationship between MBSR and the severity of depression symptoms among the HNC participants in this study. MBSR significantly decrease the severity of depression symptoms partially via declining degree of internalized stigma.

In term of the domains of internalized stigma as mediating factors between the relationship between MBSR and the severity of anxiety symptoms, MBSR exerted a significant effect on SWA (*F* [1,107] = 22.623, *R*^2^ = 0.175, *p*  < 0.001), SSC (*F* [1,107] = 8.148, *R*^2^ = 0.071, *p*  < 0.001) and SS (*F* [1,107] = 14.049, *R*^2^ = 0.116, *p*  < 0.001), whereby MBSR significantly decrease SWA (*p*  < 0.001), SSC (*p* = 0.005), and SS (*p*  < 0.001). However, MBSR did not exert significant effect on the degree of regret (*F* [1,107] = 2.308, *R*^2^ = 0.021, *p* = 0.132). In addition, MBSR, SWA, SSC, and regret exerted significant effect on the severity of anxiety symptoms (*F* [5,103] = 31.803, *R*^2^ = 0.607, *p*  < 0.001), in which SWA (*p* = 0.015), SSC (*p* = 0.006), and regret (*p* = 0.002) significantly increase the degree of anxiety symptoms, but SS did not (*p* = 0.780). The direct effect of MBSR on the severity of anxiety symptoms was significant (*B* = −3.011, 95% CI = −5.101 to 2.433, *p*  < 0.001) but it was less than that of the total effect (*B* = −5.255, 95% CI = −5.101 to 2.433, *p*  < 0.001). While the total indirect effects of SWA, SSC, SS, and regret on the relationship between MBSR and severity of anxiety symptoms was significant (*B* = −1.848, 95% CI = −3.166 to 0.653), however only SWA (*B* = −0.961, SE = 0.554, 95% CI = −2.180 to 0.046), and SSC (*B* = −0.643, SE = 0.367, 95% CI = −1.501 to −0.056) exerted significant indirect effect, not SS (*B* = 0.071, 95% CI = −0.445 to 0.633) and regret (B = −0.316, 95% CI = −0.820 to 0.110). In conclusion, the findings indicated that SWA and SSC exerted a partial mediation effect onto the relationship between MBSR and the severity of anxiety symptoms among the HNC participants in this study. MBSR significantly decrease the severity of anxiety symptoms partially via declining degrees of SWA and SSC.

In term of the domains of internalized stigma as mediating factors between the relationship between MBSR and the severity of depression symptoms, MBSR exerted a significant effect on SWA (*F* [1,107] = 22.623, *R*^2^ = 0.175, *p*  < 0.001), SSC (*F* [1,107] = 8.148, *R*^2^ = 0.071, *p*  < 0.001), and SS (*F* [1,107] = 14.049, *R*^2^ = 0.116, *p*  < 0.001), whereby MBSR significantly decrease SWA (*p*  < 0.001), SSC (*p* = 0.005), and SS (*p*  < 0.001). However, MBSR did not exert significant effect on the degree of regret (*F* [1,107] = 2.308, *R*^2^ = 0.021, *p* = 0.132). In addition, MBSR, SWA, SSC, and regret exerted significant effect on the severity of depression symptoms (*F* [5,103] = 34.075, *R*^2^ = 0.623, *p*  < 0.001), in which SWA (*p* = 0.024), SSC (*p* = 0.019), and regret (*p*  < 0.001) significantly increase the degree of depression symptoms, but SS did not (*p* = 0.831). The direct effect of MBSR on the severity of depression symptoms was significant (*B* = −3.155, 95% CI = −5.101 to 2.433, *p*  < 0.001) but it was less than that of the total effect (*B* = −5.509, 95% CI = −5.101 to 2.433, *p*  < 0.001). While the total indirect effects of SWA, SSC, SS, and regret on the relationship between MBSR and severity of depression symptoms was significant (*B* = −1.872, 95% CI = −3.275 to 0.667), however only SSC (*B* = −0.565, 95% CI = −1.389 to 0.044) exerted significant indirect effect, not SWA (B = −0.917, 95% CI = −2.152 to 0.040), SS (*B* = 0.056, 95% CI = −0.441 to 0.747), and regret (*B* = −0.447, 95% CI = −1.178 to 0.106). In conclusion, the findings indicated that speech and social concerns (SSC) exerted a partial mediation effect onto the relationship between MBSR and the severity of depression symptoms among the HNC participants in this study. MBSR significantly decrease the severity of depression symptoms partially via declining degree of SSC.

## 4. Discussion

This RCT successfully investigated the effects of MBSR on the severity of depression and anxiety symptoms, as well as the degree of internalized stigma and its domains, across three timepoints among HNC patients. Additionally, it evaluated the mediation effects of internalized stigma and its domains on the relationship between MBSR and the severity of depression and anxiety symptoms. Our findings revealed that MBSR significantly reduced the severity of depression and anxiety symptoms at all three timepoints (from *T*_0_ to *T*_1_, from *T*_1_ to *T*_2_, and maintaining the overall reduction from *T*_0_ to *T*_2_). In contrast, participants in the TAU group exhibited an overall increase in the severity of depression and anxiety symptoms over the same period (from *T*_0_ to *T*_2_). Similarly, MBSR significantly reduced the degree of internalized stigma and three of its domains—SWA, SSCs, and SS—across all three timepoints (from *T*_0_ to *T*_1_, from *T*_1_ to *T*_2_, and maintaining the overall reduction from *T*_0_ to *T*_2_). However, the degree of regret showed a delayed response, starting to decline only from *T*_1_ to *T*_2_, and maintaining the overall reduction from *T*_0_ to *T*_2_. Moreover, the effects of MBSR on reducing the severity of depression and anxiety symptoms were partially mediated by its impact on decreasing the degree of internalized stigma. Specifically, the effect of MBSR on reducing anxiety severity was partially mediated by its impact on reducing SWA and SSCs. Similarly, the effect of MBSR on reducing depression severity was partially mediated by its impact on reducing SSCs.

The effectiveness of MBSR in reducing the severity of anxiety and depression symptoms among cancer patients is well-documented in a meta-analysis that included 16 studies with a total of 2072 breast cancer patients [31]. However, data on the effectiveness of MBSR for HNC patients are rarely reported. Our findings clearly indicate that MBSR is also effective in alleviating the severity of depression and anxiety symptoms among HNC patients. MBSR involves practices like the body scan, which enables participants to focus on and attend to the physical discomfort triggered by anxiety symptoms. When coupled with awareness, acceptance, and acknowledgment of bodily sensations, this practice facilitates reflective coping. Essentially, MBSR mimics exposure therapy by guiding participants to face uncomfortable physical stimuli without avoiding them, preventing hyper-emotional responses. This process allows anxiety symptoms to gradually dissolve over time. In addition, practices such as sitting meditation, where participants maintain a seated position for an extended period and control the urge to move [32], help participants develop a better sense of physical control. This, in turn, further alleviates their anxiety symptoms and may explain the effectiveness of MBSR in reducing the severity of anxiety symptoms among HNC patients in this study. Notably, this study also found that in the absence of MBSR, as seen in the TAU control group, participants' severity of anxiety symptoms continued to escalate over time.

According to the MMT, MBSR operates by initially inducing attentional focus, which allows a shift away from negative thoughts and emotions toward the present state of self and the surrounding environment. This process facilitates decentering, which helps eliminate and dissipate working memory related to stress appraisal, attentional biases linked to stressors, maladaptive behaviors, and disruptive cognitive schemas, ultimately inducing a state of metacognitive awareness. Decentering helps individuals focus on both internal sensations and the external environment, enhancing awareness of previously unattended interoceptive and exteroceptive sensations. This process promotes positive reappraisal of trauma or the severe stress associated with living with cancer and its treatments. In turn, it induces positive affect and a sense of meaningfulness, even when living with cancer, which helps dissipate depression symptoms among the HNC participants in this study [10]. As a result, participants in the MBSR group exhibited lower HADS depression scores across time. In contrast, participants in the TAU group, who did not receive any active psychological intervention, showed an increase in the severity of depression symptoms over time.

Previous studies have not investigated the effectiveness of MBSR in reducing the degree of internalized stigma among HNC patients. However, MBSR has been reported to reduce internalized stigma over time in other cancer populations, such as breast and lung cancer patients [33]. Our study extends these findings by demonstrating that MBSR can also decrease the degree of internalized stigma among HNC patients. This effect can be explained through the MMT. Initially, MBSR facilitates attentional focus and decentering, shifting attention away from the negative and unpleasant thoughts, emotions, and behaviors induced by the severe stress of living with cancer and its treatments. It instead promotes heightened awareness of internal sensations and the external environment, creating a metacognitive state of awareness. This awareness encourages the focus on interoceptive and exteroceptive sensations and fosters the development of positive reappraisal of the severe stressor. Over time, this process induces a sense of meaningfulness in life and positive affectivity, which contributes to the reduction in internalized stigma [10, 34].

Regarding the effect of MBSR on the domains of internalized stigma, this study found that MBSR reduced the degrees of SWA, SSCs, sense of stigma, and regret over time. Theoretically, mindfulness can be understood through three interrelated mechanisms: intention of mindfulness practice (which is carried out on purpose), attention (involves paying attention to the present self and surrounding environment), and attitude (to adopt an attitude with openness and to accept any thoughts, feelings and perception in a nonjudgmental way). These mechanisms may lead to a process of “re-perceiving,” where individuals re-evaluate their inner experiences and gain a better understanding of their personal challenges, strengths, and contributions. Through self-regulation and nonjudgmental acceptance of one's own experiences (nonreactive awareness), mindfulness facilitates decentering and “re-perceiving.” This process is likely to enhance self-compassion [35]. Higher self-compassion, in turn, is associated with reduced SWA and a decreased SS among cancer patients [36, 37]. Additionally, group-based MBSR, such as the intervention in this study, may foster a sense of belonging through communication among members, enabling patients to redefine themselves through observation, learning, and gaining social support. MBSR also enhances acceptance of painful thoughts, emotions, and behaviors associated with cancer, allowing patients to share their experiences with family members and spouses, which strengthens their social support network [37]. Consequently, the positive impact of MBSR on social support likely contributed to the reduction of SSCs among HNC patients in this study. Moreover, as MBSR enhance self-compassion through decentering and group MBSR facilitates social connectedness, MBSR may attenuate internalized stigma through full mediation by social connectedness or partial mediation of social connectedness and self-compassion [38]. Similarly, MBSR facilitation of decentering and “re-perceiving” may help cancer patients to accept and nonjudgmentally acknowledge regrets related to unfinished tasks or unfulfilled goals in life [34]. This process likely contributed to the reduction of regret among HNC patients in this study.

The partial mediating effect of internalized stigma on the relationship between MBSR and the severity of depression and anxiety symptoms among HNC participants in this study can be explained based on the MMT. MBSR facilitates attentional focus and decentering by shifting attention toward previously unattended interoceptive and exteroceptive sensations, as well as the external environment, thereby, forming a metacognitive state of awareness. This shift of focus away from the unpleasant reappraisal of stress stimuli, attentional bias toward stressors, and maladaptive cognitive schemas and behaviors helps promote positive reappraisal of living with cancer and the side effects of its treatment. As a result, internalized stigma dissipates, and positive affectivity is induced, which helps counteract the severity of depression and anxiety symptoms.

Further assessment of the components of internalized stigma revealed that the effect of MBSR on reducing the degree of SSCs which partially mediated its impact on reducing the severity of both depression and anxiety symptoms among HNC participants. This highlights the pivotal role of MBSR in group psychotherapy and its contribution to this benefit. It underscores the importance of enhancing social support among cancer patients, particularly through improved communication with their family and spouses [39], as a possible mechanism underlying the effectiveness of MBSR in alleviating depression and anxiety symptoms in cancer patients. Additionally, this study found that the MBSR effect on reducing the degree of SWA which also partially mediated the reduction in the severity of anxiety symptoms among HNC participants, emphasizing the role of MBSR in enhancing self-compassion in cancer patients [35]. However, the MBSR effect on reducing the degree of SS and regret did not mediate the relationship between MBSR and the severity of anxiety and depression symptoms, indicating that lowering these two components of internalized stigma does not contribute to the mechanism underlying the effectiveness of MBSR in reducing depression and anxiety symptoms among HNC patients.

There are several limitations to consider in this study. First, the gender and ethnicity distribution of the study sample was not representative of the broader HNC population in Malaysia. Our sample included a relatively larger proportion of female and Malay patients compared to the general HNC population in the country [40]. Therefore, the findings may not be fully generalizable to the entire HNC population in Malaysia. Second, the treatment modalities received by the participants were not assessed in this study. The type of treatment received by cancer patients could be an important confounding factor that may affect primary outcomes, such as the severity of depression and anxiety symptoms [28]. Third, the follow-up assessment at the third timepoint (12 weeks after the completion of the intervention) was relatively short. Ideally, the follow-up should have been conducted at 6 months after the completion of the intervention. However, some RCTs of the effects of MBSR on stigma related to cancer, depression, and anxiety showed that MBSR exerted significant effects even at 12 weeks of follow-up [15, 16, 41].

Despite these limitations, this study provides valuable data on the effectiveness of 8-week group MBSR in ameliorating the degree of internalized stigma in HNC patients, as well as the mediating effects of internalized stigma and its components on the relationship between MBSR and the severity of anxiety and depression symptoms, which are scarce. This study also offers essential information for clinicians and researchers, suggesting a possible mechanism underlying the efficacy of MBSR in treating depression and anxiety among HNC patients through its effects on reducing the degrees of shame related to physical appearance, and SSCs. Based on the study findings, MBSR may be indicated for cancer patients with: (1) anxiety disorders related to physical disfigurement and body image issues as MBSR reduce SS and shame due to appearance, (2) depression and/or anxiety disorder due to concern with social interaction related to the cancer itself or adverse effects of its treatment as MBSR improve social connectedness and social interaction, (3) issues of cancer treatment compliance and barrier in cancer treatment seeking behavior due to internalized stigma of having cancer as MBSR alleviate cancer-related stigma, and (4) poor social support and network due to sense of cancer-related stigma that hinders the attainment of unmet needs related to cancer. The recommended resources and training needed to integrate MBSR into the treatment regimen in the oncology settings include: (1) establish collaboration with cancer centers and oncology departments in the hospital setting, (2) training of MBSR therapists or instructors would required enrollment in an 8-week MBSR program with 2.5 h per group session per week and engaging in silent retreat for basic knowledge of conducting MBSR sessions as the minimal requirement. Further training in intensive MBSR course may need another 2 to 3 years to complete, (3) materials to guide patients on learning MBSR such as workbook and home assignments, handouts, MBSR guide VCD and other meditation or yoga audiovisual recordings, (4) rating scales or screening tools to monitor the progress of MBSR such as the HADS, the 7-item generalized anxiety disorder scale, the Mindful Attention Awareness Scale, the SSS, and the Self-Compassion Scale, (5) quiet and comfortable rooms for MBSR group sessions within the cancer centers or oncology department, and (6) experienced MBSR therapists for supervision of the training sessions of the MBSR instructors. In the context of cost-effectiveness of integrating MBSR into cancer treatment regimen in the oncology settings, instead of an 8-week MBSR program, an alternative 6-week MBSR program can be implemented in which its effectiveness for treatment of depression and anxiety among cancer patients has been documented [42, 43]. However, the effectiveness of a 6-week MBSR program on ameliorating internalized stigma among cancer patients remain unprecedented and would warrant further investigation in future study.

Similarly, the question of whether MBSR effects on self-compassion and social support play a role in the partial mediating effects of decreasing SWA, and SSCs on the relationship between MBSR and the severity of depression and anxiety symptoms among HNC patients remains unexplored and warrants further investigation.

## 5. Conclusion

This study provides new insights into the effectiveness of MBSR in alleviating prevalent psychological complications associated with HNC, such as depression and anxiety. Given its positive effects on reducing depression and anxiety symptoms, as well as internalized stigma and its components, MBSR should be recommended as part of the treatment regimen for HNC patients, with benefits lasting at least 12 weeks after the intervention. Furthermore, since MBSR appears to reduce depression and anxiety symptoms by lowering the degrees of SWA and SSCs, further research should explore the effectiveness of other MBIs (such as mindfulness-based cognitive therapy and mindfulness-based cancer recovery) in addressing SWA and SSC in cancer patients.

## Figures and Tables

**Table 1:** The sociodemographic and clinical characteristics of all the participants.

Variables	Number of participants (*n*)		Percentage (%)		*p*-Value
	MBSR group	Control group	MBSR group	Control group	
Age					
18–45 years	24	22	43.6	40.0	—
≥46 years	31	33	56.4	60.0	0.847
Gender					
Male	29	27	52.7	49.1	—
Female	26	28	47.3	50.9	0.849
Ethnicity					
Malays	29	39	52.7	70.9	—
Nonmalays	26	16	47.3	29.1	0.077
Marital status					
Married	45	46	81.8	83.6	—
Nonmarried	10	9	18.2	16.4	1.000
Types of head and neck cancer					
NPC	29	21	52.7	38.2	—
Others	26	34	47.3	61.8	0.180
Stage of cancer					
Stages 1 and 2	15	16	27.3	29.1	—
Stages 3 and 4	40	39	72.7	70.9	1.000
Time since diagnosis					
New case	19	19	34.5	34.5	—
1–6 Months	36	36	65.5	65.5	1.000

**Table 2:** The main effect of between-subject factor (intervention groups), within-subject factor (timepoints), and the interaction between between-subject factor (intervention groups) and within-subject factor (timepoints) of the HADS (depression) subscale, HADS (anxiety) subscale, and total SSS scores as the dependent variable following intention-to-treat, per protocol and last observation carry forward analyses.

Dependent variables (analysis)	Numerator degree of freedom	Denominator degree of freedom	*F*	*p*-Value
HADS depression (intention-to-treat analysis)				
Between MBSR and TAU groups	1	112	13.727	<0.001*⁣*^*∗*^
Within timepoints *t*_0_, *t*_1_, and *t*_2_	2	206	7.986	<0.001*⁣*^*∗*^
Interaction of between and within-subject factors	2	206	31.575	<0.001*⁣*^*∗*^
HADS depression (per-protocol analysis)				
Between MBSR and TAU groups	1	102	15.934	<0.001*⁣*^*∗*^
Within timepoints *t*_0_, *t*_1_, and *t*_2_	2	196	12.631	<0.001*⁣*^*∗*^
Interaction of between and within-subject factors	2	196	34.063	<0.001*⁣*^*∗*^
HADS depression (last observation bring forward analysis)				
Between MBSR and TAU groups	1	111	15.750	<0.001*⁣*^*∗*^
Within timepoints *t*_0_, *t*_1_, and *t*_2_	2	214	8.965	<0.001*⁣*^*∗*^
Interaction of between and within-subject factors	2	214	36.541	<0.001*⁣*^*∗*^
HADS anxiety (intention-to-treat analysis)				
Between MBSR and TAU groups	1	114	0.927	0.338
Within timepoints *t*_0_, *t*_1_, and *t*_2_	2	208	3.956	0.044*⁣*^*∗*^
Interaction of between and within-subject factors	2	208	32.591	<0.001*⁣*^*∗*^
HADS anxiety (per-protocol analysis)				
Between MBSR and TAU groups	1	104	0.606	0.438
Within timepoints *t*_0_, *t*_1_, and *t*_2_	2	198	5.606	0.004*⁣*^*∗*^
Interaction of between and within-subject factors	2	198	33.791	<0.001*⁣*^*∗*^
HADS anxiety (last observation bring forward analysis)				
Between MBSR and TAU groups	1	113	1.327	0.252
Within timepoints *t*_0_, *t*_1_, and *t*_2_	2	216	3.677	0.027*⁣*^*∗*^
Interaction of between and within-subject factors	2	216	37.329	<0.001*⁣*^*∗*^
Total SSS (intention-to-treat analysis)				
Between MBSR and TAU groups	1	115	22.286	<0.001*⁣*^*∗*^
Within timepoints *t*_0_, *t*_1_, and *t*_2_	2	212	5.791	0.004*⁣*^*∗*^
Interaction of between and within-subject factors	2	212	12.744	<0.001*⁣*^*∗*^
Total SSS (per-protocol analysis)				
Between MBSR and TAU groups	1	105	21.183	<0.001*⁣*^*∗*^
Within timepoints *t*_0_, *t*_1_, and *t*_2_	2	202	9.570	<0.001*⁣*^*∗*^
Interaction of between and within-subject factors	2	202	13.570	<0.001*⁣*^*∗*^
Total SSS (last observation bring forward analysis)				
Between MBSR and TAU groups	1	114	22.836	<0.001*⁣*^*∗*^
Within timepoints *t*_0_, *t*_1_, and *t*_2_	2	220	7.249	<0.001*⁣*^*∗*^
Interaction of between and within-subject factors	2	220	14.530	<0.001*⁣*^*∗*^

*⁣* ^*∗*^ *p*  < 0.05.

**Table 3:** The post hoc between group comparison of the HADS depression, HADS anxiety, and total SSS scores between the MBSR and TAU control groups in each timepoints (*T*_0_, *T*_1_, and *T*_2_) after adjusted for confounding factors (age, gender, types of head and neck cancer, and time since diagnosis) following intention-to-treat analysis.

Intention-to-treat analysis (timepoints)	Mean HADS depression score in MBSR (SD), sample size (*n*)	Mean HADS depression score in TAU (SD), sample size (*n*)	Adjusted mean difference (95% confidence interval)	*p*-Value	SMD
*T*_0_	8.42 (4.16), 55	7.93 (3.87), 55	0.582 (−0.850–2.014)	0.424	0.122
*T*_1_	5.45 (2.58), 53	8.74 (4.31), 53	−3.176 (−4.620 to 1.732)	<0.001*⁣*^*∗*^	−0.926
*T*_2_	4.52 (2.64), 52	9.24 (4.58), 51	−4.588 (−6.048 to 3.127)	<0.001*⁣*^*∗*^	−1.263

**Intention-to-treat analysis (timepoints)**	**Mean HADS anxiety score in MBSR (SD), sample size (*n*)**	**Mean HADS anxiety score in TAU (SD), sample size (*n*)**	**Adjusted mean difference (95% confidence interval)**	***p*-Value**	**SMD**

*T*_0_	9.24 (3.50), 55	7.02 (3.12), 55	2.255 (0.962–3.548)	<0.001*⁣*^*∗*^	0.670
*T*_1_	7.02 (2.63), 53	8.32 (3.86), 53	−1.275 (−2.580–0.030)	0.055	−0.394
*T*_2_	5.96 (2.66), 52	8.65 (4.12), 51	−2.651 (−3.971 to 1.331)	<0.001*⁣*^*∗*^	−0.776

**Intention-to-treat analysis (timepoints)**	**Mean total SSS score in MBSR (SD), sample size (*n*)**	**Mean total SSS score in TAU (SD), sample size (*n*)**	**Adjusted mean difference (95% confidence interval)**	***p*-Value**	**SMD**

*T*_0_	24.38 (14.17), 55	32.75 (16.11), 55	−7.820 (−13.785 to 1.854)	0.011*⁣*^*∗*^	−0.552
*T*_1_	19.23 (11.03), 53	33.72 (21.00), 53	−13.450 (−19.445 to 7.456)	<0.001*⁣*^*∗*^	−0.864
*T*_2_	14.62 (7.07), 52	35.22 (20.73),51	−18.990 (−25.029 to 12.951)	<0.001*⁣*^*∗*^	−1.201

*Note*: *T*_0_ = baseline assessment prior to intervention, *T*_1_ = 8 weeks after intervention commenced or immediately after completion of intervention), and *T*_2_ = 12 weeks after completion of intervention.

Abbreviations: MBSR, mindfulness-based stress reduction; SMD, standardized mean difference; TAU, treatment-as-usual controls.

*⁣* ^*∗*^Statistical significance at *p*  < 0.05.

**Table 4:** Post hoc within group comparison of the changes in the HADS depression, HADS anxiety, and total SSS scores across timepoints in the MBSR and the TAU control groups after adjusted for confounding factors (age, gender, types of head and neck cancer, and time since diagnosis) following intention-to-treat analysis.

Intervention group	Adjusted mean difference between timepoints (95% CI)	Standard error	*p*-Value	SMD
HADS (depression)-intention-to-treat analysis				
MBSR	*T*_0_ to *T*_1_: −2.877 (−3.787 to −1.967)	0.377	<0.001*⁣*^*∗*^	−0.858
	*T*_1_ to *T*_2_: −0.934 (−1.852 to −0.015)	0.381	0.045*⁣*^*∗*^	−0.356
	*T*_0_ to *T*_2_: −3.811 (−5.004 to −2.617)	0.495	<0.001*⁣*^*∗*^	−1.119
TAU	*T*_0_ to *T*_1_: 0.881 (−0.029 to 1.791)	0.377	0.061	0.198
	*T*_1_ to *T*_2_: 0.478 (−0.449 to 1.404)	0.384	0.644	0.116
	*T*_0_ to *T*_2_: 1.359 (0.159 to 2.558)	0.498	0.020*⁣*^*∗*^	0.309
HADS (anxiety)-intention-to-treat analysis				
MBSR	*T*_0_ to *T*_1_: −2.194 (−3.044 to −1.344)	0.352	<0.001*⁣*^*∗*^	−0.717
	*T*_1_ to *T*_2_: −1.049 (−1.907 to −0.191)	0.356	0.011*⁣*^*∗*^	−0.401
	*T*_0_ to *T*_2_: −3.243 (−4.351 to −2.135)	0.460	<0.001*⁣*^*∗*^	−1.055
TAU	*T*_0_ to *T*_1_: 1.336 (0.486 to 2.186)	0.352	<0.001*⁣*^*∗*^	0.370
	*T*_1_ to *T*_2_: 0.327 (−0.538 to 1.193)	0.359	1.000	0.085
	*T*_0_ to *T*_2_: 1.663 (0.549 to 2.777)	0.462	0.001*⁣*^*∗*^	0.446
Total SSS-intention-to-treat analysis				
MBSR	*T*_0_ to *T*_1_: −4.895 (−7.657 to −2.134)	1.144	<0.001*⁣*^*∗*^	−0.406
	*T*_1_ to *T*_2_: −4.430 (−7.217 to −1.643)	1.155	<0.001*⁣*^*∗*^	−0.498
	*T*_0_ to *T*_2_: −9.325 (−13.088 to −5.562)	1.561	<0.001*⁣*^*∗*^	−0.864
TAU	*T*_0_ to *T*_1_: 0.736 (−2.026 to 3.497)	1.144	1.000	0.052
	*T*_1_ to *T*_2_: 1.109 (−1.703 to 3.922)	1.166	1.000	0.072
	*T*_0_ to *T*_2_: 1.845 (−1.937 to 5.627)	1.144	0.722	0.002

*Note*: *T*_0_ = baseline assessment prior to intervention, *T*_1_ = 8 weeks after intervention commenced or immediately after completion of intervention), and *T*_2_ = 12 weeks after completion of intervention.

Abbreviations: MBSR, mindfulness-based stress reduction; SMD, standardized mean difference; TAU, treatment-as-usual controls.

*⁣* ^*∗*^Statistical significance at *p* < 0.05.

**Table 5:** The main effect of between-subject factor (intervention groups), within-subject factor (timepoints), and the interaction between between-subject factor (intervention groups) and within-subject factor (timepoints) of the domains of SSS scores (as the dependent variable) following intention-to-treat, per-protocol, and last observation carry forward analyses.

Dependent variables (analysis)	Numerator degree of freedom	Denominator degree of freedom	*F*	*p*-Value
Shame with appearance (intention-to-treat analysis)				
Between MBSR and TAU groups	1	116	12.999	<0.001*⁣*^*∗*^
Within timepoints *t*_0_, *t*_1_, and *t*_2_	2	211	3.386	0.044*⁣*^*∗*^
Interaction of between and within-subject factors	2	211	11.190	<0.001*⁣*^*∗*^
Shame with appearance (per-protocol analysis)				
Between MBSR and TAU groups	1	103	12.350	<0.001*⁣*^*∗*^
Within timepoints *t*_0_, *t*_1_, and *t*_2_	2	117	4.280	0.016*⁣*^*∗*^
Interaction of between and within-subject factors	2	117	10.280	<0.001*⁣*^*∗*^
Shame with appearance (last observation bring forward analysis)				
Between MBSR and TAU groups	1	115	13.054	<0.001*⁣*^*∗*^
Within timepoints *t*_0_, *t*_1_, and *t*_2_	2	220	3.794	0.043*⁣*^*∗*^
Interaction of between and within-subject factors	2	220	12.000	<0.001*⁣*^*∗*^
Speech and social concern (intention-to-treat analysis)				
Between MBSR and TAU groups	1	113	25.675	<0.001*⁣*^*∗*^
Within timepoints *t*_0_, *t*_1_, and *t*_2_	2	209	3.322	0.038*⁣*^*∗*^
Interaction of between and within-subject factors	2	209	4.160	0.017*⁣*^*∗*^
Speech and social concern (per-protocol analysis)				
Between MBSR and TAU groups	1	103	26.949	<0.001*⁣*^*∗*^
Within timepoints *t*_0_, *t*_1_, and *t*_2_	2	200	6.309	0.002*⁣*^*∗*^
Interaction of between and within-subject factors	2	200	4.020	0.019*⁣*^*∗*^
Speech and social concern (last observation bring forward analysis)				
Between MBSR and TAU groups	1	113	26.876	<0.001*⁣*^*∗*^
Within timepoints *t*_0_, *t*_1_, and *t*_2_	2	218	4.322	0.014*⁣*^*∗*^
Interaction of between and within-subject factors	2	218	5.332	0.005*⁣*^*∗*^
Sense of stigma (intention-to-treat analysis)				
Between MBSR and TAU groups	1	115	16.352	<0.001*⁣*^*∗*^
Within timepoints *t*_0_, *t*_1_, and *t*_2_	2	210	3.110	0.047*⁣*^*∗*^
Interaction of between and within-subject factors	2	210	7.654	<0.001*⁣*^*∗*^
Sense of stigma (per-protocol analysis)				
Between MBSR and TAU groups	1	105	13.515	<0.001*⁣*^*∗*^
Within timepoints *t*_0_, *t*_1_, and *t*_2_	2	201	4.711	0.010*⁣*^*∗*^
Interaction of between and within-subject factors	2	201	7.672	<0.001*⁣*^*∗*^
Sense of stigma (last observation bring forward analysis)				
Between MBSR and TAU groups	1	114	16.463	<0.001*⁣*^*∗*^
Within timepoints *t*_0_, *t*_1_, and *t*_2_	2	219	3.745	0.025*⁣*^*∗*^
Interaction of between and within-subject factors	2	219	8.031	<0.001*⁣*^*∗*^
Regret (intention-to-treat analysis)				
Between MBSR and TAU groups	1	115	20.542	<0.001*⁣*^*∗*^
Within timepoints *t*_0_, *t*_1_, and *t*_2_	2	209	1.541	0.217
Interaction of between and within-subject factors	2	209	3.810	0.042*⁣*^*∗*^
Regret (per-protocol analysis)				
Between MBSR and TAU groups	1	105	18.815	<0.001*⁣*^*∗*^
Within timepoints *t*_0_, *t*_1_, and *t*_2_	2	199	2.871	0.059
Interaction of between and within-subject factors	2	199	3.714	0.026*⁣*^*∗*^
Regret (last observation bring forward analysis)				
Between MBSR and TAU groups	1	114	16.463	<0.001*⁣*^*∗*^
Within timepoints *t*_0_, *t*_1_, and *t*_2_	2	219	2.745	0.065
Interaction of between and within-subject factors	2	219	8.031	<0.001*⁣*^*∗*^

*⁣* ^*∗*^ *p*  < 0.005.

**Table 6:** The post hoc between group comparison of the SSS domain scores between the MBSR and TAU control groups in each timepoints (*T*_0_, *T*_1_, and *T*_2_) after adjusted for confounding factors (age, gender, types of head and neck cancer, and time since diagnosis) following intention-to-treat analysis.

Intention-to-treat analysis (timepoints)	Mean shame with appearance score in MBSR (SD), sample size (*n*)	Mean shame with appearance score in TAU (SD), sample size (*n*)	Adjusted mean difference (95% confidence interval)	*p*-Value	SMD
*T*_0_	9.55 (5.78), 55	11.38 (6.69), 55	−1.659 (−4.153–0.835)	0.191	−0.293
*T*_1_	7.08 (4.85), 53	12.32 (8.37), 53	−4.782 (−7.291 to 2.273)	<0.001*⁣*^*∗*^	−0.766
*T*_2_	6.19 (4.01), 52	12.98 (8.29), 51	−6.227 (−8.758 to 3.696)	<0.001*⁣*^*∗*^	−1.043

**Intention-to-treat analysis (timepoints)**	**Mean speech and social concern score in MBSR (SD), sample size (*n*)**	**Mean speech and social concern score in TAU (SD), sample size (*n*)**	**Adjusted mean difference (95% confidence interval)**	***p*-Value**	**SMD**

*T*_0_	3.29 (3.13), 55	5.69 (2.95), 55	−2.340 (−3.564 to 1.116)	<0.001*⁣*^*∗*^	−0.789
*T*_1_	2.60 (2.51), 53	5.57 (3.73),53	−2.796 (−4.026 to 1.565)	<0.001*⁣*^*∗*^	−0.934
*T*_2_	1.88 (2.44), 52	5.82 (3.93), 51	−3.668 (−4.908 to 2.427)	<0.001*⁣*^*∗*^	−1.205

**Intention-to-treat analysis (timepoints)**	**Mean sense of stigma score in MBSR (SD), sample size (*n*)**	**Mean sense of stigma score in TAU (SD), sample size (*n*)**	**Adjusted mean difference (95% confidence interval)**	***p*-Value**	**SMD**

*T*_0_	5.78 (4.94), 55	8.00 (5.54), 55	−1.943 (−3.963–0.077)	0.059	−0.423
*T*_1_	3.89 (3.83), 53	8.49 (6.89), 53	−4.246 (−6.279 to 2.212)	<0.001*⁣*^*∗*^	−0.825
*T*_2_	3.00 (2.99), 52	8.76 (7.10), 51	−5.170 (−7.223 to 3.117)	<0.001*⁣*^*∗*^	−1.057

**Intention-to-treat analysis (timepoints)**	**Mean regret score in MBSR (SD), sample size (*n*)**	**Mean regret score in TAU (SD), sample size (*n*)**	**Adjusted mean difference (95% confidence interval)**	***p*-Value**	**SMD**

*T*_0_	5.76 (2.27), 55	7.67 (3.12), 55	−1.912 (−2.930 to 0.895)	<0.001*⁣*^*∗*^	−0.700
*T*_1_	5.66 (1.72), 53	7.34 (3.44), 53	−1.685 (−2.711 to 0.659)	0.001*⁣*^*∗*^	−0.618
*T*_2_	4.98 (2.06), 52	7.65 (3.05), 51	−2.615 (−3.655 to 1.574)	<0.001*⁣*^*∗*^	−1.026

*Note*: *T*_0_ = baseline assessment prior to intervention, *T*_1_ = 8 weeks after intervention commenced or immediately after completion of intervention), and *T*_2_ = 12 weeks after completion of intervention.

Abbreviations: MBSR, mindfulness-based stress reduction; SMD, standardized mean difference; TAU, treatment-as-usual controls.

*⁣* ^*∗*^Statistical significance at *p*  < 0.05.

**Table 7:** Post hoc within group comparison of the changes in the SSS domain scores across timepoints in the MBSR and the TAU control groups after adjusted for confounding factors (age, gender, types of head and neck cancer, and time since diagnosis) following intention-to-treat analysis.

Intervention group	Adjusted mean difference between timepoints (95% CI)	Standard error	*p*-Value	SMD
Shame with appearance (intention-to-treat analysis)				
MBSR	*T*_0_ to *T*_1_: −2.366 (−3.655 to −1.077)	0.534	<0.001*⁣*^*∗*^	−0.463
	*T*_1_ to *T*_2_: −0.828 (−2.129 to 0.473)	0.539	0.379	−0.200
	*T*_0_ to *T*_2_: −3.194 (−4.931 to −1.456)	0.721	<0.001*⁣*^*∗*^	−0.675
TAU	*T*_0_ to *T*_1_: 0.757 (−0.532 to 2.046)	0.534	0.474	0.124
	*T*_1_ to *T*_2_: 0.617 (−0.695 to 1.930)	0.544	0.773	0.079
	*T*_0_ to *T*_2_: 1.374 (−0.372 to 3.121)	0.725	0.177	0.212
Speech and social concerns (intention-to-treat analysis)				
MBSR	*T*_0 to_ *T*_1_: −0.587 (−1.186 to 0.012)	0.517	0.057	−0.243
	*T*_1_ to *T*_2_: −0.682 (−1.286 to −0.078)	0.517	0.021*⁣*^*∗*^	−0.291
	*T*_0_ to *T*_2_: −1.269 (−2.080 to −0.457)	0.696	<0.001*⁣*^*∗*^	−0.502
TAU	*T*_0_ to *T*_1_: −0.131 (−0.730 to 0.467)	0.517	1.000	−0.036
	*T*_1_ to *T*_2_: 0.190 (−0.419 to 0.800)	0.517	1.000	0.065
	*T*_0_ to *T*_2_: 0.059 (−0.757 to 0.875)	0.696	1.000	0.037
Sense of stigma (intention-to-treat analysis)				
MBSR	*T*_0_ to *T*_1_: −1.859 (−2.988 to −0.731)	0.467	<0.001*⁣*^*∗*^	−0.428
	*T*_1_ to *T*_2_: −0.806 (−1.945 to 0.332)	0.472	0.267	−0.259
	*T*_0_ to *T*_2_: −2.666 (−4.173 to −1.159)	0.625	<0.001*⁣*^*∗*^	−0.681
TAU	*T*_0_ to *T*_1_: 0.443 (−0.685 to 1.571)	0.467	1.000	0.078
	*T*_1_ to *T*_2_: 0.118 (−1.031 to 1.267)	0.476	1.000	0.039
	*T*_0_ to *T*_2_: 0.561 (−0.954 to 2.076)	0.629	1.000	0.119
Regret (intention-to-treat analysis)				
MBSR	*T*_0_ to *T*_1_: −0.093 (−0.754 to 0.567)	0.274	1.000	−0.050
	*T*_1_ to *T*_2_: −0.704 (−1.377 to −0.032)	0.279	0.037*⁣*^*∗*^	−0.358
	*T*_0_ to *T*_2_: −0.798 (−1.665 to 0.070)	0.360	0.083	−0.360
TAU	*T*_0_ to *T*_1_: −0.320 (−0.981 to 0.340)	0.274	0.729	−0.100
	*T*_1_ to *T*_2_: 0.225 (−0.447 to 0.898)	0.279	1.000	0.095
	*T*_0_ to *T*_2_: −0.095 (−0.963 to 0.772)	0.360	1.000	−0.006

*Note*: *T*_0_ = baseline assessment prior to intervention, *T*_1_ = 8 weeks after intervention commenced or immediately after completion of intervention), and *T*_2_ = 12 weeks after completion of intervention.

Abbreviations: MBSR, mindfulness-based stress reduction; SMD, standardized mean difference; TAU, treatment-as-usual controls.

*⁣* ^*∗*^Statistical significance at *p* < 0.05.

**Table 8:** The mediation effect of total SSS and its domains on the relationship between MBSR and HADS anxiety subscale score.

Mediators	Path	Coefficient	SE	*β*	*t*	*p*	Bootstrapping (LLCI to ULCI)
Total SSS	A	−11.527	2.287	−0.869	−5.040	<0.001*⁣*^*∗*^	−16.061 to −6.994
	B	0.195	0.026	0.546	7.599	<0.001*⁣*^*∗*^	0.144 to 0.245
	C	−5.255	0.752	−1.111	−6.987	<0.001*⁣*^*∗*^	−6.745 to −3.764
	C'	−3.011	0.677	−0.637	−4.449	<0.001*⁣*^*∗*^	−4.352 to −1.669
	A × B	−2.244	0.590	—	—	—	−3.493 to −1.181
	A × B (partially standardized indirect effect [*β*])	−0.474	0.108	—	—	—	−0.699 to −0.276

SWA	a	−4.624	0.972	−0.832	−4.756	<0.001*⁣*^*∗*^	−6.551 to −2.697
	b	0.208	0.084	0.243	2.484	0.015*⁣*^*∗*^	0.042 to 0.374
	C	−5.255	0.752	−1.111	−6.987	<0.001*⁣*^*∗*^	−6.745 to −3.764
	C'	−3.011	0.677	−0.637	−4.449	<0.001*⁣*^*∗*^	−4.352 to −1.669
	a × b	−0.961	0.554	—	—	—	−2.183 to −0.041
	a × b (partially standardized indirect effect [*β*])	−0.202	0.111	—	—	—	−0.435 to −0.009

SSC	a'	−1.462	0.512	−0.530	−2.854	0.005*⁣*^*∗*^	−2.478 to −0.447
	b'	0.439	0.157	0.255	2.798	0.006*⁣*^*∗*^	0.128 to 0.751
	C	−5.255	0.752	−1.111	−6.987	<0.001*⁣*^*∗*^	−6.745 to −3.764
	C'	−3.011	0.677	−0.637	−4.449	<0.001*⁣*^*∗*^	−4.352 to −1.669
	a' × b'	−0.643	0.363	—	—	—	−1.465 to −0.071
	a' × b' (partially standardized indirect effect [*β*])	−0.135	0.074	—	—	—	−0.299 to −0.015

SS	a”	−3.362	0.897	−0.678	−3.748	<0.001*⁣*^*∗*^	−5.140 to −1.584
	b”	−0.021	0.076	−0.022	−0.280	0.780	−0.172 to 0.129
	C	−5.255	0.752	−1.111	−6.987	<0.001*⁣*^*∗*^	−6.745 to −3.764
	C'	−3.011	0.677	−0.637	−4.449	<0.001*⁣*^*∗*^	−4.352 to −1.669
	a” × b”	0 0.071	0.264	—	—	—	−0.450 to 0 0.633
	a” × b” (partially standardized indirect effect [*β*])	0 0.015	0.055	—	—	—	−0.094 to 0.129

Regret	a”'	−0.799	0.526	−0.289	−1.519	0.132	−1.84 to 0.244
	b”'	0.396	0.122	0.230	3.248	0.002*⁣*^*∗*^	0.154 to 0.637
	C	−5.255	0.752	−1.111	−6.987	<0.001*⁣*^*∗*^	−6.745 to −3.764
	C'	−3.011	0.677	−0.637	−4.449	<0.001*⁣*^*∗*^	−4.352 to −1.669
	a”' × b”'	−0.316	0.236	—	—	—	−0.816 to 0.124
	a”' × b”' (partially standardized indirect effect [*β*])	−0.067	0.048	—	—	—	−0.165 to 0.027

Abbreviations: SS, sense of stigma; SSC, social and speech concern; SSS, Shame and Stigma Scale; SWA, shame of appearance.

***⁣* ^*∗*^** Statistical significance set at *p*  < 0.05.

**Table 9:** The mediation effect of total SSS and its domains on the relationship between MBSR and HADS depression subscale score.

Mediators	Path	Coefficient	SE	*β*	*t*	*p*	Bootstrapping (LLCI to ULCI)
Total SSS	A	−11.527	2.287	−0.869	−5.040	<0.001*⁣*^*∗*^	−16.061 to −6.994
	B	0.204	0.027	0.542	7.474	<0.001*⁣*^*∗*^	0.150 to 0.258
	C	−5.509	0.798	−1.102	−6.907	<0.001*⁣*^*∗*^	−7.090 to −3.928
	C'	−3.155	0.722	−0.631	−4.370	<0.001*⁣*^*∗*^	−5.101 to −2.433
	A × B	−2.354	0.587	—	—	—	−3.554 to −1.263
	A × B (partially standardized indirect effect [*β*])	−0.471	0.101	—	—	—	−0.672 to −0.278

SWA	a	−4.624	0.972	−0.832	−4.756	<0.001*⁣*^*∗*^	−6.551 to −2.697
	b	0.198	0.086	0.220	2.298	0.024*⁣*^*∗*^	0.027 to 0.369
	C	−5.509	0.798	−1.102	−6.907	<0.001*⁣*^*∗*^	−7.090 to −3.928
	C'	−3.155	0.722	−0.631	−4.370	<0.001*⁣*^*∗*^	−5.101 to −2.433
	a × b	−0.917	0.566	—	—	—	−2.150 to 0.061
	a	−0.183	0.109	—	—	—	−0.413 to 0.013

SSC	a'	−1.462	0.512	−0.530	−2.854	0.005*⁣*^*∗*^	−2.478 to −0.447
	b'	0.387	0.162	0.213	2.387	0.019*⁣*^*∗*^	0.065 to 0.708
	C	−5.509	0.798	−1.102	−6.907	<0.001*⁣*^*∗*^	−7.090 to −3.928
	C'	−3.155	0.722	−0.631	−4.370	<0.001*⁣*^*∗*^	−5.101 to −2.433
	a' × b'	−0.565	0.338	—	—	—	−1.351 to −0.061
	a' × b' (partially standardized indirect effect [*β*])	−0.113	0.066	—	—	—	−0.264 to −0.013

SS	a”	−3.362	0.897	−0.678	−3.748	<0.001*⁣*^*∗*^	−5.140 to −1.584
	b”	−0.017	0.078	−0.017	−0.214	0.831	−0.172 to 0.139
	C	−5.509	0.798	−1.102	−6.907	<0.001*⁣*^*∗*^	−7.090 to −3.928
	C'	−3.155	0.722	−0.631	−4.370	<0.001*⁣*^*∗*^	−5.101 to −2.433
	a” × b”	0.056	0.308	—	—	—	−0.463 to 0.775
	a” × b” (partially standardized indirect effect [*β*])	0.011	0.061	—	—	—	−0.093 to 0.153

Regret	a”'	−0.799	0.526	−0.289	−1.519	0.132	−1.841 to 0.244
	b”'	0.559	0.126	0.309	4.455	<0.001*⁣*^*∗*^	0.310 to 0.808
	C	−5.509	0.798	−1.102	−6.907	<0.001*⁣*^*∗*^	−7.090 to −3.928
	C'	−3.155	0.722	−0.631	−4.370	<0.001*⁣*^*∗*^	−5.101 to −2.433
	a”' × b”'	−0.447	0.329	—	—	—	−1.156 to 0.128
	a”' × b”' (partially standardized indirect effect [*β*])	−0.089	0.063	—	—	—	−0.221 to 0.028

Abbreviations: SS, sense of stigma; SSC, social and speech concern; SSS, Shame and Stigma Scale; SWA, shame of appearance.

***⁣* ^*∗*^** Statistical significance set at *p*  < 0.05.

## Data Availability

The data that support the findings of this study are available from the corresponding author upon reasonable request.

## Supporting Information

Supporting Information 1 File 1. The detailed description of the MBSR manual used as guideline in this study.

Supporting Information 2 File 2. The CONSORT checklist for this study.

Supporting Information 3 Table S1. The post hoc between group comparison of the HADS depression, HADS anxiety, and total SSS scores between the MBSR and TAU control groups in each timepoint (*T*_0_, *T*_1_, and *T*_2_) after adjusted for confounding factors (age, gender, types of head and neck cancer, and time since diagnosis), following per-protocol and last observation carry forward analyses.

Supporting Information 4 Table S2. The post hoc between group comparison of the SSS domain scores between the MBSR and TAU control groups in each timepoint (*T*_0_, *T*_1_, and *T*_2_) after adjusted for confounding factors (age, gender, types of head and neck cancer, and time since diagnosis), following per-protocol and last observation carry forward analyses.

Supporting Information 5 Table S3. Post hoc within group comparison of the changes in the HADS depression, HADS anxiety, and total SSS scores across timepoints in the MBSR and the TAU control groups after adjusted for confounding factors (age, gender, types of head and neck cancer, and time since diagnosis) following per-protocol and last observation carry forward analyses.

Supporting Information 6 Table S4. Post hoc within group comparison of the changes in the SSS domain scores across timepoints in the MBSR and the TAU control groups after adjusted for confounding factors (age, gender, types of head and neck cancer, and time since diagnosis) following per-protocol and last observation carry forward analyses.
