# Supplementary material for: Mindfulness-Based Stress Reduction Alleviates Depression, Anxiety, and Internalized Stigma Compared With Treatment-as-Usual Among Head and Neck Cancer Patients: Findings From a Randomized Controlled Trial
Source: Depress Anxiety. 2025 Sep 11;2025:7499120. doi: 10.1155/da/7499120 (PMC12446601; doi:10.1155/da/7499120)
Supplement: Supporting Information 1 — File 1. The detailed description of the MBSR manual used as guideline in this study. [file 7499120.f1.pdf]

Supplementary file 1. The detailed description of the MBSR manual used in this study

MBSR were performed as group sessions, with each session lasting 2.5 hours. These sessions took place once a week over a 6-week period, accompanied by an additional 45 minutes of daily home practice. The structure of the MBSR sessions followed the therapy format established by Kabat-Zinn (Kabat-Zinn, 2003).

(i) Session 1: Waking up from automatic pilot.

In the beginning of this session, the therapist discussed and explained to respondents about mindfulness-based therapy and the importance of home practice. Spending time to do home practice was a challenging part of this course but the outcomes were worth doing. Then, the therapist did the automatic pilot activity. This activity aimed to increase awareness, then, we could respond to the situation by choice and not reacted automatically. In the automatic pilot, we tended to dimly aware of what we were doing and follow habits of thinking which were unhelpful and led to stress. The therapist would do a 'raisin exercise' to show how our attention was not always placed fully in the present moment, and to explore how attention could change experiences.

After that, the therapist performed a 'body scan' in half an hour. In the body scan exercise, respondents were encouraged to place attention on the different parts of the body and used each part of our body as the anchor of our awareness at the moment, which emphasized that when became more aware of the present moment, our thoughts feelings and bodily sensations, we could give ourselves greater freedom and choice. In the meantime, we further increased participants' awareness and feelings about automatic pilot by reminding them to pay attention to whether their attention was distracted as the body scan performing. Mindfulness was not about trying to get anywhere but being aware of where and how we were allowing ourselves to be that.

Before the end of the session, we would request participants to carry out a daily home practice. The first one was to keep repeating a body scan for 10 to 20 minutes daily, for which we would remind the participants to give up all expectations about and just let the experience be the experience. The second home practice was to choose a routine activity in the daily life and made a deliberate effort to bring full awareness to that activity each time. The final assignment was named '10-finger gratitude practice', which meant counting ten pleasure events that happened in the daytime and brought you positive experiences.

(ii) Session 2: Keeping the body in mind.

Firstly, we engaged in a new meditation exercise: sitting with breath. Focusing on the sensation of breath through different parts of our physical body was a quick pathway to keep the body in mind. For example, to perceive the breath through the rise and fall of the chest and abdomen. Besides, once we got in touch with our breathing, we could also notice how the breath changed the moods, thoughts and even body movements. With practice, participants could use breath as an anchor of the body bringing sensation of safe, calm and pace, and also used breath to relax tense muscle, deal with pain, anger, relationships or the stress of daily life.

For the second part of this session, we performed a metaphorical approach by imagining a scenario where our participants encountered a friend on the street, and showed greetings to the friend, turned out being ignored by the friend. Then our therapists defused the experiences of participants into emotions, thoughts, behavioral impulses and also body feelings. Through sharing the outcomes among group members, we guided participants to draw the conclusion: thoughts were so important to be aware of because it is the thoughts which decide emotions, behavioral impulses, and affect body feelings. According to this, insisting home practice to keep body in mind was very effective to perceive the thoughts.

The last part was named '50:50 attention', for which we taught participants using breath as an anchor, and kept some attention on it while in conversations. Being awareness at moment was helpful to get away from automatic pilot and enabled making more appropriate responses.

After the intervention, we would encourage participants to keep a pleasure experiences calendar (**Table 3.1**) as home practice, for which they chose a pleasurable experience daily and defused them into emotions, thoughts, behavioral impulse and body feelings. In addition to this, '50:50 attention' and body scan were also recommended to continue practicing as the habits in life.

Table 3.1 Experiences Calendar

Day	What was the pleasant experience?	How did your body Feel in detail during this experience?	What moods and feelings accompanied the experience?	What thoughts went through your mind?	What impulses or behaviors did you notice?
Day 1					
Day 2					
Day 3					
Day 4					
Day 5					
Day 6					

(iii) Session 3: Revisiting session 1 and 2

In the third MBSR session, the therapist will review the assignments and lessons of Session 2, practice sitting meditations, and collect feedback from respondents regarding this session. The therapist will practice three-minute breathing exercises with respondents and give respondents home assignments regarding what was revisited in this session.

(iv) Session 4: Gathering the scattered mind.

In this session, the therapist would firstly review the home practices, doing 5-min exercises of sitting with breath. Then, our therapists taught participants to keep mindfulness of body in movement, some yoga and mindful stretching were designed for this part on order to explore the limitations of the physical body as well. Then, the therapist explained that the bodily discomfort we almost inevitably encountered when practice mindful stretching provided an ideal situation to learn how to approach the difficult and unwanted experiences with curiosity, gentleness, kindness and courage. During movement, it was also a time when attention was most prone to scattering, through this process, it was even more beneficial for training participants' focus and concentration.

After that, a new practice to gather the scattered mind was trained, we named it as '3-step breathing space'. First step was becoming aware, which used body scan or breathing practice to bring thoughts, feelings, and body sensations into consciousness to avoid automatic processing. The second step was gathering, whereby the focus of attention is towards the physical sensation experience during breathing. This step will focus the mind on the breathing, wherever the sensation is experienced most vividly. Hence, the emphasis is on the sensations which are experienced in a full duration in-breath and full duration out-breath. Finally, the last step involves extension of the field of awareness around the breath to include the whole body as well as the posture and facial expression. The breathing exercise offered an opportunity to get away from automatic pilot and reestablish a connection with the present. With each instance of practicing the breathing exercise, we become conscious of our breath and the sensations we are experiencing. Before ending of this session, we encouraged participants to continue the experiences calendar as home practice, but instead of pleasure events, we focused on unpleasant events dairy in this week. In addition, 3-step breathing space and mindfulness movement were also required as daily practice.

(v) Session 5: Recognizing reactivity.

The therapists started the session by reviewing the assignments and lessons learned in the previous session. Then we trained our participants to sit observing their thoughts and reactivities. Usually, metaphor was commonly used in this part, as example we got used to guide our participants to imagine the thoughts as leaves in the river or the screens of cinema, and then performed themselves as the bystander, just watching without controlling.

We also used 'The Vicious Flower' (**Fig.3.1**) to show the reactivities of us, when we experienced pain or distress in the mind which often took a route that seemed to offer a way out but in fact kept difficulties going or makes them worse. That's because the reactivities were often attempting to control or escape from these unpleasant experiences. Conversely, acceptance and allowing them could be a better choice. In that case, we would require the participants to perform a '3-step breathing space' before they reacted to one thing, which was efficient to make a wise response.

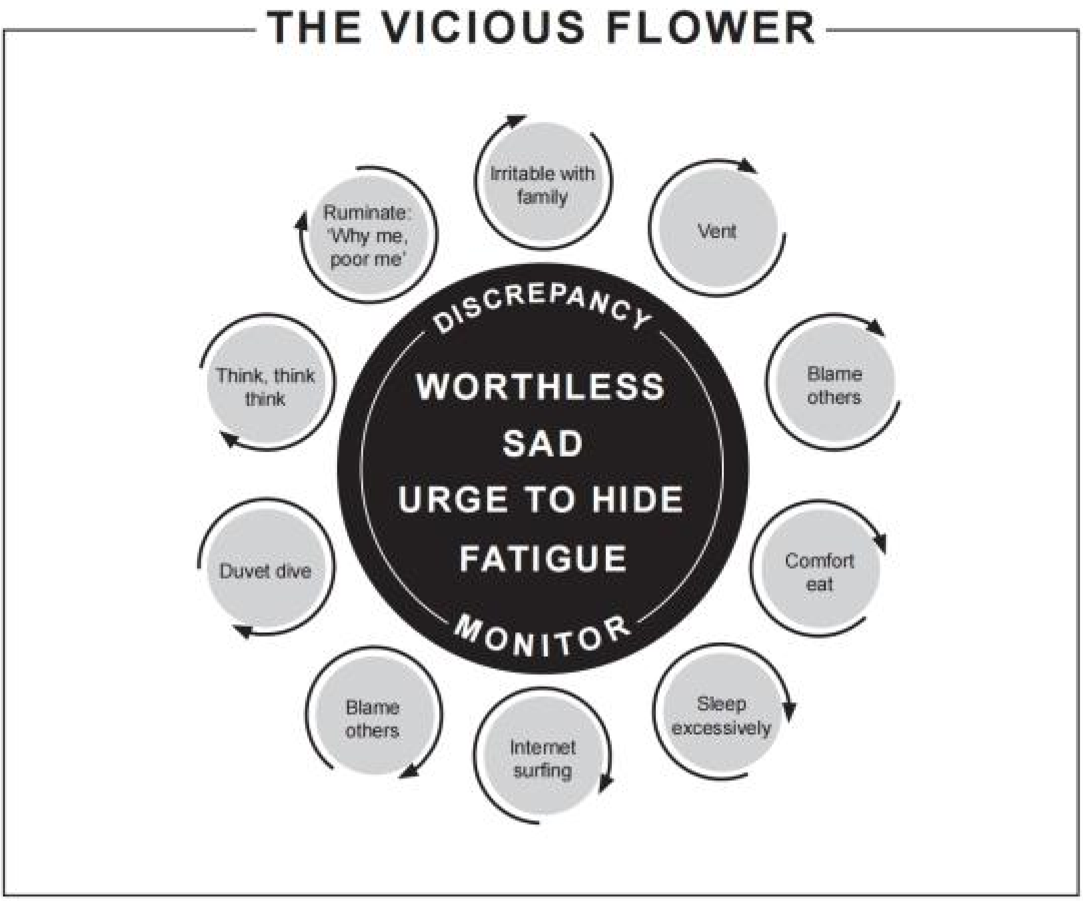

Fig. 3.1 Conception of Vicious Flower

By the end of this session, we took a review of the techniques learned before and suggested to our participants to choose some as the home practice for this week.

(vi) Session 6: Revisiting session 4 and 5

During the sixth MBSR session, the therapist will discuss the previous session's lesson and encourage respondents to use their bodies to increase their awareness. By remaining aware of the present moment, they can mindfully respond instead of automatically reacting to stressors. However, such responses require that respondents experience and accept stressors in the present moment. Then, respondents will walk mindfully and perform a three-minute breathing space exercise and recognizing reactivities through 'The Vicious Flower'.

(vii) Session 7: Thoughts are not facts.

In this session, the therapist would discuss again the values determined by the respondent from the previous session. Then we led our participants to practice sitting with observing thoughts and reactivities, to enhance their ability to make the wise responses. In the meantime, '3-step breathing space' was combined to this part.

After that, we used an imagination practice to accelerate the further understanding of our thoughts. We led our participants to imagine two different scenarios. In one, they sought help from colleagues after receiving criticism from their supervisor during a morning meeting, but their request was declined from their colleagues. In the other, it was the same situation of

seeking assistance from colleagues, but this time it followed praise from the supervisor during the meeting. Through these two scenarios, we guided participants to experience how their thoughts and emotions might differ, followed by group sharing. The events that unfold were the same – seeking help from colleagues and being declined. However, based on different contexts, they often led to different thoughts. Additionally, the thoughts of different participants could vary even in response to the same event. According to this, the concept that thoughts were not facts emerged. It was remarkable how liberating it felt to be able to see the thoughts were just thoughts and that they were not 'you' or 'reality'.

At the end of this session, our therapists also introduced some ways that we could see the thoughts differently. For instance, tried watching thoughts arrive and leave, without feeling that you have to follow them or viewing the thoughts as mental events rather than facts, thoughts were always representations of situations rather than being the situations themselves or writing difficult or repetitive thoughts down on paper, which let us to perceive them with lesser degrees of emotion and overwhelming experience. For particularly difficult thoughts, it may help by having another look at them intentionally, in a more balanced and opened state of mind when this is available. The home practice for this session was not specifically structured. Instead, we encouraged participants to engage in daily practice based on their personal preferences.

(viii) Session 8: How could I best take care of myself?

In the last session, the therapist summarized the content of previous sessions, and discussed programs and encourage respondents to continue mindful exercises regularly. After reconnecting with expanded awareness, we led participants to do the kindness practice, for which we guided participants to individually imagine three different kinds of people in their minds: someone they disliked, someone they liked, and themselves. By sending the same well-wishes to each of them, they experienced the sense of positivity that kindness could bring to their lives.

In addition, we invited participants to engage in a brief exercise focused on experiencing sensations, for which their daily life activities were divided into two categories: depleting activities and nourishing activities. Then, we would ask participants to make choices about what they must let go of as increasing pressures mount. Typically, participants were more inclined to give up their nourishing activities to gain time for work. But in reality, they might fall into a vicious cycle, much like an exhaustion funnel (**Fig.3.2**). The exhaustion funnel illustrated a process that easily happened to us. The narrowing area of the circles represented the narrowing of our lives that took place when we give up the things that we enjoy but that seemed 'optional'. We stopped doing activities that would nourish us, leaving only work or other stressors that often depleted our resources.

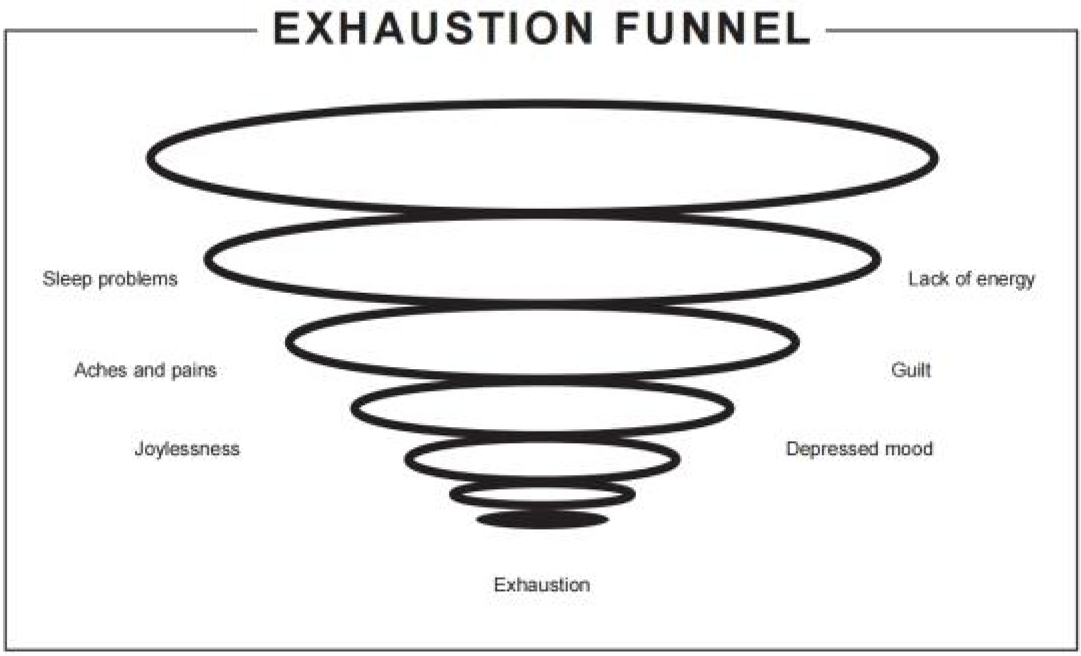

Fig.3.2 Conception of Exhaustion Funnel

Subsequently, participants would be asked the following questions: How did I spend my time? And did the choices that I made about this really supported my well-being and that of those around me?" After that, it might feel appropriate to take some considered action that supported our well-being. In response to persistent difficult thoughts and feelings, for example, the following activities might be particularly helpful: did something pleasurable, or something that would give you a sense of accomplishment or satisfaction, or acting mindfully, or choosing not to do anything.

Once all interventions were concluded, each participant would receive a workbook on the mindfulness practices. We would also conduct regular follow-ups with participants to ensure the effectiveness of the interventions.
