# Supplementary material for: Mindfulness-Based Stress Reduction Alleviates Depression, Anxiety, and Internalized Stigma Compared With Treatment-as-Usual Among Head and Neck Cancer Patients: Findings From a Randomized Controlled Trial
Source: Depress Anxiety. 2025 Sep 11;2025:7499120. doi: 10.1155/da/7499120 (PMC12446601; doi:10.1155/da/7499120)
Supplement: Supporting Information 3 — Table S1. The post hoc between group comparison of the HADS depression, HADS anxiety, and total SSS scores between the MBSR and TAU control groups in each timepoint (T0, T1, and T2) after adjusted for confounding factors (age, gender, types of head and neck cancer, and time since diagnosis), following per-protocol and last observation carry forward analyses. [file 7499120.f3.pdf]

Supplementary table 1. The post-hoc between group comparison of the HADS-Depression, HADS-Anxiety and total SSS scores between the MBSR and TAU control groups in each time points (T0, T1 and T2) after adjusted for confounding factors (age, gender, types of head and neck cancer and time since diagnosis) following per-protocol and last observation carry forward analyses

**Per-protocol analysis (time points)**	**Mean HADS-Depression score in MBSR (SD), sample size (n)**	**Mean HADS-Depression score in TAU (SD), sample size (n)**	**Adjusted mean difference (95% confidence interval)**	***p*-value**	**SMD**
T_0	8.63 (4.16), 51	8.04 (4.15), 50	0.591 (-0.845 to 2.026)	0.417	0.142
T_1	5.42 (2.56), 51	8.72 (4.04), 50	-3.285 (-4.720 to -1.849)	< 0.001*	-0.976
T_2	4.30 (2.63), 51	9.24 (4.31), 50	-4.942 (-6.378 to -3.507)	< 0.001*	-1.384
**Last observation carry forward analyses (time points)**	**Mean HADS-Depression score in MBSR (SD), sample size (n)**	**Mean HADS-Depression score in TAU (SD), sample size (n)**	**Adjusted mean difference (95% confidence interval)**	***p*-value**	**SMD**
T_0	8.42 (4.16), 55	7.93 (4.15), 55	0.584 (-0.843 to 2.012)	0.420	0.118
T_1	5.50 (2.58), 55	8.79 (4.29), 55	-3.288 (-4.716 to -1.861)	< 0.001*	-0.929
T_2	4.42 (2.64), 55	9.35 (4.48), 55	-4.925 (-6.352 to -3.497)	< 0.001*	-1.341
**Per-protocol analysis (time points)**	**Mean HADS-Anxiety score in MBSR (SD), sample size (n)**	**Mean HADS-Anxiety score in TAU (SD), sample size (n)**	**Adjusted mean difference (95% confidence interval)**	***p*-value**	**SMD**
T_0	9.59 (3.50), 51	7.15 (3.12), 50	2.440 (1.077 to 3.803)	< 0.001*	0.736
T_1	7.27 (2.56), 51	8.27 (3.86), 50	-0.994 (-2.357 to 0.369)	0.152	-0.305
T_2	5.82 (2.82), 51	8.67 (4.03), 50	-2.865 (-4.228 to	< 0.001*	-0.819

			-1.502)		

Last observation carry forward analyses (time points)	Mean HADS-Anxiety score in MBSR (SD), sample size (n)	Mean HADS-Anxiety score in TAU (SD), sample size (n)	Adjusted mean difference (95% confidence interval)	*p*-value	SMD
T_0	9.36 (3.50), 55	7.08 (3.12), 55	2.275 (0.971 to 3.579)	< 0.001*	0.688
T_1	7.12 (2.58), 55	8.43 (3.89), 55	-1.307 (-2.611 to -0.002)	0.050	-0.397
T_2	5.87 (2.75), 55	8.85 (4.13), 55	-2.979 (-4.284 to -1.675)	< 0.001*	-0.849

Per-protocol analysis (time points)	Mean total SSS score in MBSR (SD), sample size (n)	Mean total SSS score in TAU (SD), sample size (n)	Adjusted mean difference (95% confidence interval)	*p*-value	SMD
T_0	25.48 (14.17), 51	33.06 (16.11), 50	-7.585 (-13.606 to -1.564)	0.014*	-0.500
T_1	20.24 (10.83), 51	32.98 (20.68), 50	-12.741 (-18.762 to -6.720)	< 0.001*	-0.772
T_2	14.93 (6.85), 51	33.98 (19.89), 50	-19.054 (-25.075 to -13.033)	< 0.001*	-1.281

Last observation carry forward analyses (time points)	Mean total SSS score in MBSR (SD), sample size (n)	Mean total SSS score in TAU (SD), sample size (n)	Adjusted mean difference (95% confidence interval)	*p*-value	SMD
T_0	25.20 (14.17), 55	32.99 (16.11), 55	-7.786 (-13.713 to -1.859)	0.010*	-0.513
T_1	20.34 (10.95), 55	33.63 (20.68), 55	-13.277 (-19.204 to -7.350)	< 0.001*	-1.891
T_2	15.37 (6.90), 55	34.68 (21.92),	-19.313 (-25.240	< 0.001*	-1.188

55	to -<br>13.386)	

* statistical significance at $p < 0.05$, T_0 = baseline assessment prior to intervention, T_1 = 8 weeks after intervention commenced (immediately after completion of intervention), T_2 = 12 weeks after completion of intervention, MBSR = mindfulness based stress reduction, TAU = treatment-as-usual controls, SMD = standardized mean difference
