# Supplementary material for: Mindfulness-Based Stress Reduction Alleviates Depression, Anxiety, and Internalized Stigma Compared With Treatment-as-Usual Among Head and Neck Cancer Patients: Findings From a Randomized Controlled Trial
Source: Depress Anxiety. 2025 Sep 11;2025:7499120. doi: 10.1155/da/7499120 (PMC12446601; doi:10.1155/da/7499120)
Supplement: Supporting Information 4 — Table S2. The post hoc between group comparison of the SSS domain scores between the MBSR and TAU control groups in each timepoint (T0, T1, and T2) after adjusted for confounding factors (age, gender, types of head and neck cancer, and time since diagnosis), following per-protocol and last observation carry forward analyses. [file 7499120.f4.pdf]

Supplementary table 2. The post-hoc between group comparison of the SSS domain scores between the MBSR and TAU control groups in each time points (T0, T1 and T2) after adjusted for confounding factors (age, gender, types of head and neck cancer and time since diagnosis) following per-protocol and last observation carry forward analyses

Per-protocol analysis (time points)	Mean Shame with appearance score in MBSR (SD), sample size (n)	Mean Shame with appearance score in TAU (SD), sample size (n)	Adjusted mean difference (95% confidence interval)	*p*-value	SMD
T_0	9.94 (5.78), 51	11.63 (6.69), 50	-1.691 (-4.202 to 0.820)	0.185	-0.270
T_1	7.41 (4.73), 51	11.99 (8.09), 50	-4.502 (-7.013 to -1.991)	< 0.001*	-0.691
T_2	6.41 (3.81), 51	12.57 (7.91), 50	-6.160 (-8.672 to -3.649)	< 0.001*	-0.992

Last observation carry forward analyses (time points)	Mean Shame with appearance score in MBSR (SD), sample size (n)	Mean Shame with appearance score in TAU (SD), sample size (n)	Adjusted mean difference (95% confidence interval)	*p*-value	SMD
T_0	9.90 (5.78), 55	11.55 (6.69), 55	-1.644 (-4.126 to 0.838)	0.193	-0.231
T_1	7.57 (4.82), 55	12.07 (8.32), 55	-4.662 (-7.144 to -2.180)	< 0.001*	-0.662
T_2	6.56 (3.89), 55	12.69 (8.14) 55	-6.298 (-8.780 to -3.816)	< 0.001*	-0.961

Per-protocol analysis (time points)	Mean Speech and social concerns score in MBSR (SD), sample size (n)	Mean Social concern score in TAU (SD), sample size (n)	Adjusted mean difference (95% confidence interval)	*p*-value	SMD
T_0	3.25 (3.13), 51	5.72 (2.95), 50	-2.475 (-3.707 to -1.242)	< 0.001*	-0.812
T_1	2.64 (2.53), 51	5.46 (3.52), 50	-2.823 (-4.055 to -1.590)	< 0.001*	-0.920
T_2	1.78 (2.43), 51	5.50 (3.65), 50	-3.725 (-4.958 to	< 0.001*	-1.200

			-2.493)		
**Last observation carry forward analyses (time points)**	**Mean Speech and social concerns score in MBSR (SD), sample size (n)**	**Mean Social concern score in TAU (SD), sample size (n)**	**Adjusted mean difference (95% confidence interval)**	***p*-value**	**SMD**
T_0	3.36 (3.13), 55	5.69 (2.95), 55	-2.334 (-3.547 to -1.120)	< 0.001*	-0.789
T_1	2.78 (2.52), 55	5.57 (3.66), 55	-2.788 (-4.002 to -1.575)	< 0.001*	-0.907
T_2	1.96 (2.38), 55	5.73 (3.84), 55	-3.770 (-4.983 to -2.557)	< 0.001*	-1.202
**Per-protocol analysis (time points)**	**Mean Sense of stigma score in MBSR (SD), sample size (n)**	**Mean Sense of stigma score in TAU (SD), sample size (n)**	**Adjusted mean difference (95% confidence interval)**	***p*-value**	**SMD**
T_0	6.47 (4.94), 51	8.15 (5.54), 50	-1.673 (-3.745 to 0.398)	0.113	-0.320
T_1	4.55 (3.75), 51	8.39 (6.84), 50	-3.835 (-5.906 to -1.763)	< 0.001*	-0.608
T_2	3.47 (2.93), 51	8.51 (6.91), 50	-5.033 (-7.105 to -2.962)	< 0.001*	-0.899
**Last observation carry forward analyses (time points)**	**Mean Sense of stigma score in MBSR (SD), sample size (n)**	**Mean Sense of stigma score in TAU (SD), sample size (n)**	**Adjusted mean difference (95% confidence interval)**	***p*-value**	**SMD**
T_0	6.09 (4.94), 55	8.02 (5.54), 55	-1.936 (-3.941 to 0.069	0.058	-0.368
T_1	4.27 (3.78), 55	8.44 (6.77), 55	-4.173 (-6.178 to -2.167)	< 0.001*	-0.761
T_2	3.32 (2.92), 55	8.49 (6.94), 55	-5.173 (-7.178 to -3.167)	< 0.001*	-0.971

**Per-protocol analysis (time points)**	**Mean Regret score in MBSR (SD), sample size (n)**	**Mean Regret score in TAU (SD), sample size (n)**	**Adjusted mean difference (95% confidence interval)**	***p*-value**	**SMD**
T_0	5.74 (2.27), 51	7.54 (3.12), 50	-1.799 (-2.863 to -0.735)	0.001*	-0.660
T_1	5.48 (1.74), 51	7.12 (3.41), 50	-1.634 (-2.698 to -0.570)	0.003*	-0.607
T_2	4.65 (2.21), 51	7.38 (3.01), 50	-2.732 (-3.798 to -1.665)	< 0.001*	-1.034
**Last observation carry forward analyses (time points)**	**Mean Regret score in MBSR (SD), sample size (n)**	**Mean Regret score in TAU (SD), sample size (n)**	**Adjusted mean difference (95% confidence interval)**	***p*-value**	**SMD**
T_0	5.80 (2.27), 55	7.70 (3.12), 55	-1.900 (-2.920 to -0.880)	< 0.001*	-0.696
T_1	5.67 (1.69), 55	7.35 (3.38), 55	-1.682 (-2.702 to -0.662)	0.001*	-0.629
T_2	4.82 (2.15), 55	7.57 (3.04), 55	-2.750 (-3.773 to -1.728)	< 0.001*	-1.044

* statistical significance at $p < 0.05$, T_0 = baseline assessment prior to intervention, T_1 = 8 weeks after intervention commenced (immediately after completion of intervention), T_2 = 12 weeks after completion of intervention, MBSR = mindfulness based stress reduction, TAU = treatment-as-usual controls, SMD = standardized mean difference
