# Supplementary material for: Mindfulness-Based Stress Reduction Alleviates Depression, Anxiety, and Internalized Stigma Compared With Treatment-as-Usual Among Head and Neck Cancer Patients: Findings From a Randomized Controlled Trial
Source: Depress Anxiety. 2025 Sep 11;2025:7499120. doi: 10.1155/da/7499120 (PMC12446601; doi:10.1155/da/7499120)
Supplement: Supporting Information 5 — Table S3. Post hoc within group comparison of the changes in the HADS depression, HADS anxiety, and total SSS scores across timepoints in the MBSR and the TAU control groups after adjusted for confounding factors (age, gender, types of head and neck cancer, and time since diagnosis) following per-protocol and last observation carry forward analyses. [file 7499120.f5.pdf]

Supplementary table 3. Post-hoc within group comparison of the changes in the HADS-Depression, HADS-Anxiety and total SSS scores across time points in the MBSR and the TAU control groups after adjusted for confounding factors (age, gender, types of head and neck cancer and time since diagnosis) following per-protocol and last observation carry forward analyses

Intervention group	Adjusted mean difference between time points (95% CI)	Standard error	*p*-value	SMD
	**HADS (Depression)-Per-protocol analysis**			
MBSR	T_0 to T_1: -2.314 (-3.180 to -1.447)	0.359	< 0.001*	-0.929
	T_1 to T_2: -1.451 (-2.318 to -0.584)	0.359	< 0.001*	-0.432
	T_0 to T_2: -3.765 (-4.894 to -2.636)	0.468	< 0.001*	-1.244
TAU	T_0 to T_1: 1.120 (0.245 to 1.995)	0.363	0.007*	0.123
	T_1 to T_2: 0.420 (-0.455 to 1.295)	0.363	0.744	0.121
	T_0 to T_2: 1.540 (0.400 to 2.680)	0.473	0.004*	0.278
	**HADS (Depression)-Last observation carry forward analysis**			
MBSR	T_0 to T_1: -2.877 (-3.787 to -1.967)	0.377	< 0.001*	-0.844
	T_1 to T_2: -1.073 (-1.962 to -0.183)	0.369	0.012*	-0.414
	T_0 to T_2: -4.036 (-5.200 to -2.873)	0.483	< 0.001*	-1.148
TAU	T_0 to T_1: 0.881 (-0.029 to 1.791)	0.377	0.061	0.204
	T_1 to T_2: 0.564 (-0.326 to 1.453)	0.369	0.383	0.128
	T_0 to T_2: 1.473 (0.309 to 2.636)	0.483	0.008*	0.342
	**HADS (Anxiety)-Per-protocol analysis**			
MBSR	T_0 to T_1: -2.314 (-3.180 to -1.447)	0.359	< 0.001*	-0.757
	T_1 to T_2: -1.451 (-2.318 to -0.584)	0.359	< 0.001*	-0.538
	T_0 to T_2: -3.765 (-4.894 to -2.636)	0.468	< 0.001*	-1.186
TAU	T_0 to T_1: 1.120 (0.245 to 1.995)	0.363	0.007*	0.319
	T_1 to T_2: 0.420 (-0.455 to 1.295)	0.363	0.744	0.101
	T_0 to T_2: 1.540 (0.400 to 2.680)	0.473	0.004*	0.422

**HADS (Anxiety)-Last observation carry forward analysis**				
MBSR	T_0 to T_1: -2.236 (-3.069 to -1.403)	0.345	< 0.001*	-0.729
	T_1 to T_2: -1.255 (-2.088 to -0.422)	0.345	0.001*	-0.469
	T_0 to T_2: -3.491 (-4.576 to -2.406)	0.451	< 0.001*	-1.109
TAU	T_0 to T_1: 1.345 (0.512 to 2.178)	0.345	< 0.001*	0.383
	T_1 to T_2: 0.418 (-0.415 to 1.251)	0.345	0.681	0.102
	T_0 to T_2: 1.764 (0.678 to 2.849)	0.451	< 0.001*	0.484
**Total SSS-Per-protocol analysis**				
MBSR	T_0 to T_1: -5.235 (-8.002 to -2.468)	1.146	< 0.001*	-0.416
	T_1 to T_2: -5.314 (-8.081 to -2.547)	1.146	< 0.001*	-0.586
	T_0 to T_2: -10.549 (-14.300 to -6.798)	1.556	< 0.001*	-0.948
TAU	T_0 to T_1: -0.080 (-2.874 to 2.714)	1.158	1.000	0.004
	T_1 to T_2: 1.000 (-1.794 to 3.794)	1.158	1.000	0.049
	T_0 to T_2: 0.920 (-2.869 to 4.709)	1.571	1.000	0.051
**Total SSS-Last observation carry forward analysis**				
MBSR	T_0 to T_1: -4.855 (-7.540 to -2.169)	1.113	< 0.001*	-0.384
	T_1 to T_2: -4.982 (-7.667 to -2.296)	1.113	< 0.001*	-0.543
	T_0 to T_2: -9.836 (-13.484 to -6.189)	1.513	< 0.001*	-0.882
TAU	T_0 to T_1: 0.636 (-2.049 to 3.322)	1.113	1.000	0.035
	T_1 to T_2: 1.055 (-1.631 to 3.740)	1.113	1.000	0.049
	T_0 to T_2: 1.691 (-1.957 to 5.338)	1.513	0.795	0.088

* statistical significance at $p < 0.05$, T_0 = baseline assessment prior to intervention, T_1 = 8 weeks after intervention commenced (immediately after completion of intervention), T_2 = 12 weeks after completion of intervention, MBSR = mindfulness based stress reduction, TAU = treatment-as-usual controls, SMD = standardized mean difference
