# Supplementary material for: Mindfulness-Based Stress Reduction Alleviates Depression, Anxiety, and Internalized Stigma Compared With Treatment-as-Usual Among Head and Neck Cancer Patients: Findings From a Randomized Controlled Trial
Source: Depress Anxiety. 2025 Sep 11;2025:7499120. doi: 10.1155/da/7499120 (PMC12446601; doi:10.1155/da/7499120)
Supplement: Supporting Information 6 — Table S4. Post hoc within group comparison of the changes in the SSS domain scores across timepoints in the MBSR and the TAU control groups after adjusted for confounding factors (age, gender, types of head and neck cancer, and time since diagnosis) following per-protocol and last observation carry forward analyses. [file 7499120.f6.pdf]

Supplementary table 4. Post-hoc within group comparison of the changes in the SSS domain scores across time points in the MBSR and the TAU control groups after adjusted for confounding factors (age, gender, types of head and neck cancer and time since diagnosis) following per-protocol and last observation carry forward analyses

**Shame with appearance-Per protocol analysis**				
MBSR	T_0 to T_1: -2.451 (-3.734 to -1.168)	0.532	< 0.001*	-0.479
	T_1 to T_2: -1.078 (-2.362 to 0.205)	0.532	0.131	-0.233
	T_0 to T_2: -3.529 (-5.264 to -1.795)	0.712	< 0.001*	-0.721
TAU	T_0 to T_1: 0.360 (-0.936 to 1.656)	0.537	1.000	0.048
	T_1 to T_2: 0.580 (-0.716 to 1.876)	0.537	0.844	0.072
	T_0 to T_2: 0.940 (-0.812 to 2.692)	0.719	0.583	0.128
**Shame with appearance-Last observation carry forward analysis**				
MBSR	T_0 to T_1: -2.327 (-3.574 to -1.080)	0.517	< 0.001*	-0.437
	T_1 to T_2: -1.018 (-2.265 to 0.229)	0.517	0.150	-0.254
	T_0 to T_2: -3.345 (-5.022 to -1.669)	0.696	< 0.001*	-0.673
TAU	T_0 to T_1: 0.691 (-0.556 to 1.938)	0.517	0.548	0.069
	T_1 to T_2: 0.618 (-0.629 to 1.865)	0.517	0.699	0.008
	T_0 to T_2: 1.309 (-0.368 to 2.986)	0.696	0.183	0.153
**Speech and social concerns-Per protocol analysis**				
MBSR	T_0 to T_1: -0.255 (-0.937 to 0.427)	0.282	1.000	-0.214
	T_1 to T_2: -0.838 (-1.526 to -0.151)	0.285	0.011*	-0.347
	T_0 to T_2: -1.093 (-1.984 to -0.202)	0.370	0.010*	-0.525
TAU	T_0 to T_1: -0.420 (-1.108 to 0.268)	0.285	0.427	-0.080
	T_1 to T_2: 0.260 (-0.428 to 0.948)	0.285	1.000	0.011
	T_0 to T_2: -0.160 (-1.055 to 0.735)	0.372	1.000	-0.066
**Speech and social concerns-Last observation carry forward analysis**				
MBSR	T_0 to T_1: -0.582 (-1.167 to 0.004)	0.243	0.052	-0.204

	T_1 to T_2: -0.181 (-1.404 to -0.233)	0.243	0.003*	-0.335
	T_0 to T_2: -1.400 (-2.191 to -0.609)	0.328	< 0.001*	-0.504
TAU	T_0 to T_1: -0.127 (-2.191 to -0.609)	0.243	1.000	-0.036
	T_1 to T_2: 0.164 (-0.422 to 0.749)	0.243	1.000	0.042
	T_0 to T_2: 0.036 (-0.755 to 0.827)	0.328	1.000	0.036
**Sense of stigma-Per protocol analysis**				
MBSR	T_0 to T_1: -0.608 (-1.207 to -0.009)	0.248	0.045*	-0.438
	T_1 to T_2: -0.863 (-1.461 to -0.264)	0.248	0.002*	-0.321
	T_0 to T_2: -1.471 (-2.278 to -0.663)	0.335	< 0.001*	-0.739
TAU	T_0 to T_1: -0.260 (-0.865 to 0.345)	0.250	0.902	-0.038
	T_1 to T_2: 0.040 (-0.565 to 0.645)	0.250	1.000	0.017
	T_0 to T_2: -0.220 (-1.036 to 0.596)	0.338	1.000	-0.057
**Sense of stigma-Last observation carry forward analysis**				
MBSR	T_0 to T_1: -0.582 (-1.167 to 0.004)	0.243	0.052	-0.414
	T_1 to T_2: -0.818 (-1.404 to -0.233)	0.243	0.003*	-0.281
	T_0 to T_2: -1.400 (-2.191 to -0.609)	0.328	< 0.001*	-0.683
TAU	T_0 to T_1: -0.127 (-2.191 to -0.609)	0.243	1.000	-0.068
	T_1 to T_2: 0.164 (-0.422 to 0.749)	0.243	1.000	0.007
	T_0 to T_2: 0.036 (-0.755 to 0.827)	0.328	1.000	0.075
**Regret-Per protocol analysis**				
MBSR	T_0 to T_1: -0.255 (-0.937 to 0.427)	0.282	1.000	-0.129
	T_1 to T_2: -0.838 (-1.526 to -0.151)	0.285	0.011*	-0.417
	T_0 to T_2: -1.093 (-1.984 to -0.202)	0.370	0.010*	-0.487
TAU	T_0 to T_1: -0.420 (-1.108 to 0.268)	0.285	0.427	-0.129
	T_1 to T_2: 0.260 (-0.428 to 0.948)	0.285	1.000	0.086

	T_0 to T_2: -0.160 (-1.055 to 0.735)	0.372	1.000	-0.052
	**Regret-Last observation carry forward analysis**			
MBSR	T_0 to T_1: -0.127 (-0.771 to 0.517)	0.267	1.000	-0.065
	T_1 to T_2: -0.850 (-1.500 to -0.201)	0.269	0.005*	-0.443
	T_0 to T_2: -0.978 (-1.822 to -0.133)	0.351	0.017*	-0.446
TAU	T_0 to T_1: -0.345 (-0.990 to 0.299)	0.267	0.591	-0.108
	T_1 to T_2: 0.218 (-0.426 to 0.862)	0.267	1.000	0.068
	T_0 to T_2: -0.127 (-0.968 to 0.714)	0.349	1.000	-0.042

* statistical significance at $p < 0.05$, T_0 = baseline assessment prior to intervention, T_1 = 8 weeks after intervention commenced (immediately after completion of intervention), T_2 = 12 weeks after completion of intervention, MBSR = mindfulness based stress reduction, TAU = treatment-as-usual controls, SMD = standardized mean difference
